# Different distribution of histone modifications in genes with unidirectional and bidirectional transcription and a role of CTCF and cohesin in directing transcription

**DOI:** 10.1186/s12864-015-1485-5

**Published:** 2015-04-15

**Authors:** Susanne Bornelöv, Jan Komorowski, Claes Wadelius

**Affiliations:** Department of Cell and Molecular Biology, Science for Life Laboratory, Uppsala University, Uppsala, SE-751 24 Sweden; Institute of Computer Science, Polish Academy of Sciences, Warsaw, 01-248 Poland; Science for Life Laboratory, Department of Immunology, Genetics and Pathology, Uppsala University, Uppsala, SE-751 08 Sweden; Current affiliation: Science for Life Laboratory, Department of Medical Biochemistry and Microbiology, Uppsala University, Uppsala, SE-751 23 Sweden

**Keywords:** Antisense transcription, CTCF, RAD21, Cohesin, CAGE, Epigenetics, Transcription factor, Histone modification

## Abstract

**Background:**

Several post-translational histone modifications are mainly found in gene promoters and are associated with the promoter activity. It has been hypothesized that histone modifications regulate the transcription, as opposed to the traditional view with transcription factors as the key regulators. Promoters of most active genes do not only initiate transcription of the coding sequence, but also a substantial amount of transcription of the antisense strand upstream of the transcription start site (TSS). This promoter feature has generally not been considered in previous studies of histone modifications and transcription factor binding.

**Results:**

We annotated protein-coding genes as bi- or unidirectional depending on their mode of transcription and compared histone modifications and transcription factor occurrences between them. We found that H3K4me3, H3K9ac, and H3K27ac were significantly more enriched upstream of the TSS in bidirectional genes compared with the unidirectional ones. In contrast, the downstream histone modification signals were similar, suggesting that the upstream histone modifications might be a consequence of transcription rather than a cause. Notably, we found well-positioned CTCF and RAD21 peaks approximately 60-80 bp upstream of the TSS in the unidirectional genes. The peak heights were related to the amount of antisense transcription and we hypothesized that CTCF and cohesin act as a barrier against antisense transcription.

**Conclusions:**

Our results provide insights into the distribution of histone modifications at promoters and suggest a novel role of CTCF and cohesin as regulators of transcriptional direction.

**Electronic supplementary material:**

The online version of this article (doi:10.1186/s12864-015-1485-5) contains supplementary material, which is available to authorized users.

## Background

The classical view of gene regulation is that transcription factors (TF) bind to enhancers and promoters. This leads to recruitment of RNA Pol II to the promoter and initiation of transcription. Another aspect of transcription is that several histone post-translational modifications are preferentially located in the promoter region of genes and are associated with gene activity [1,2]. This led to the hypothesis of the histone code [3], which suggested that gene activity is directed by the presence of histone modifications (HM). However, this theory has been debated [4]. The idea that TFs instead are the main determinants of gene activity is supported by different data e.g. by the observation that regions with inter-individual differences in chromatin marks are enriched for TF motif-disrupting single nucleotide polymorphisms (SNP) [5] and that disruptions of several motifs are associated with differences in HMs [6].

Gene transcription by RNA Pol II is a complex process involving several layers of regulation and is coupled to changes in the chromatin structure [7]. Additionally, most promoters initiate transcription in both directions from the TSS on the opposite strands [8,9]. Upstream antisense RNAs produced by this divergent transcription are often short and quickly degraded [10]. However, 10% of the protein-coding genes in the human genome have a bidirectional orientation separated by <1000 bp (1 kb) [11,12] suggesting that divergent transcription may have been evolutionary advantageous [10]. The presence of antisense transcription should therefore be considered in the analysis of gene-regulatory marks.

In an earlier study [13] we observed that the histone 3 acetylation (H3ac) signal upstream from the transcription start sites (TSS) was lower in unidirectional compared with bidirectional genes whereas both groups had approximately equal signal downstream of the TSS. This observation suggested that the upstream signal might be associated with whether a gene is bidirectionally transcribed. This was the main motivation for this study in which we carefully evaluated 98 publicly available datasets describing the genomic distribution of HMs, TFs, and RNA Pol II for any association with the transcriptional direction. Apart from HMs we have also studied many TFs including the CCCTC-binding factor (CTCF) and RAD21. CTCF is known for creating boundaries between enhancers and promoters and acting as a chromatin barrier [14]. RAD21 is a subunit of cohesin which is found at most regulatory elements [15] and has been shown to co-occur with CTCF to regulate gene expression [16] and to link regulatory regions to their targets [17].

In this study we compared bi- and unidirectional protein-coding genes with respect to HMs and TFs in the promoter region. The genes were annotated as bi- or unidirectional based on TSSs identified from cap analysis of gene expression (CAGE) data and genes retrieved from the Ensembl database. The HM and TF signals were obtained from publicly available ChIP-seq data within the ENCODE project and the comparison was done in six different cell lines. We found a significantly higher signal of the well-known HMs H3K4me3, H3K9ac, and H3K27ac upstream of the TSS of the bidirectional genes. Similarly, the TFs NELFe and TAF1 were significantly more enriched upstream of the TSS of bidirectional genes. Notably, we found well-positioned CTCF and RAD21 peaks 60-80 bp upstream of the TSS that were specific for unidirectional genes, suggesting that CTCF and cohesin are involved in directing the transcription. Supporting this idea, we showed that the CTCF signal in this peak is negatively correlated to H3K4me3, H3K9ac, and H3K27ac upstream of the TSS.

In conclusion, we have shown that TSS-specific HMs mainly occur in transcribed regions and could be a consequence of transcription. In addition, we have identified CTCF and cohesin as possible players in the direction of transcription initiation.

## Results

### Identification of bi- and unidirectional genes

**Figure 1:**
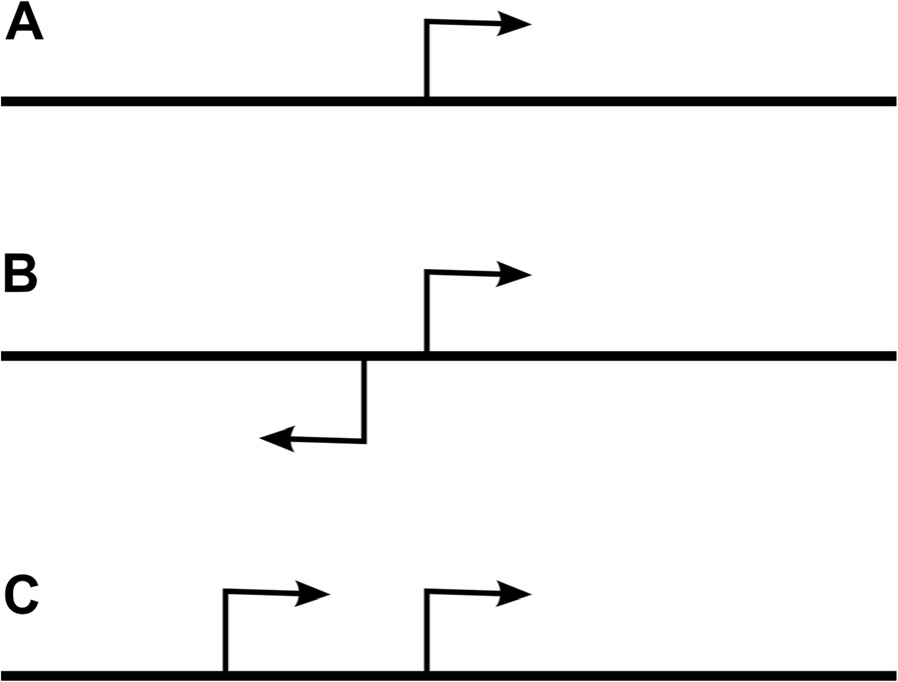
Gene promoter types. Genes were subdivided into **(A)** unidirectional, **(B)** bidirectional and **(C)** unidirectional genes with an upstream alternative TSS. The arrows represent TSSs and the figures are centered at the gene TSS.

In this study we considered different types of promoters for protein-coding genes. Many genes are unidirectional and transcribed in one direction (Figure 1A) whereas a fraction of promoters are bidirectional and initiate transcription in both directions from the different strands (Figure 1B). Some genes have alternative TSSs on the same strand (Figure 1C) and this promoter structure was also evaluated.

To identify bi- and unidirectional genes, we started from all 19,950 protein-coding genes in the Ensembl database [18]. We focused on protein-coding genes since they are sufficiently many and generally higher expressed than other types of genes, such as long non-coding RNAs [19]. The CAGE technology may be used to identify TSSs across the genome. CAGE clusters were downloaded from the ENCODE repository [20] at UCSC and filtered to contain likely promoters. Genes with a CAGE cluster nearby the TSS were defined as actively transcribed. Using this definition, the number of active genes included in the study varied between 2,839 and 6,041 for different cell lines and CAGE RNA isolation conditions (Table 1). Table 1 also provides an overview of the cell lines and datasets used for this study. For each dataset only the genes active in that cell line were included for further analysis.

**Table 1:** **Number of active genes annotated as bi- or unidirectional**

**Cell line**	**Location**	**Extract**	**Total**	**Ensembl**		**CAGE**		**Ens+CAGE**		**Agreement**
				**Bi**	**Uni**	**Bi**	**Uni**	**Bi**	**Uni**	**(%)**
GM12878	Cytosol	PolyA-	3,531	723	2,808	414	3,117	318	2,712	85.8
	Nucleolus	Total	5,244	1143	4,101	926	4,318	641	3,816	85.0
H1hESC	Cell	PolyA-	6,041	1236	4,805	889	5,152	728	4,644	88.9
HepG2	Cytosol	PolyA-	4,315	899	3,416	435	3,880	378	3,359	86.6
	Nucleolus	Total	5,169	1102	4,067	798	4,371	561	3,830	84.9
HUVEC	Cytosol	PolyA-	5,072	1107	3,965	674	4,398	603	3,894	88.7
K562	Cytosol	PolyA-	4,500	893	3,607	423	4,077	363	3,547	86.9
	Nucleolus	Total	2,839	678	2,161	411	2,428	335	2,085	85.2
NHEK	Cytosol	PolyA-	3,521	722	2,799	352	3,169	292	2,739	86.1

The number of active protein-coding genes for each cell line and RNA isolation condition (subcellular location and RNA extract). These genes were annotated as bi- or unidirectional using CAGE, Ensembl, or both methods. The agreement between the CAGE and Ensembl annotations is provided for each dataset.

**Figure 2:**
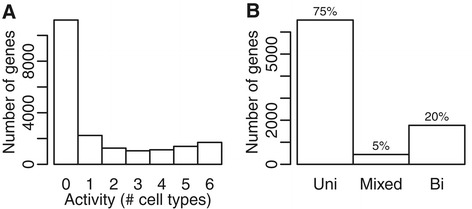
Overlap between active genes and gene annotations across cell lines. **(A)** All protein-coding genes (*n* = 19,950) were subdivided based on the number of cell lines in which they were active (between 0 and 6). **(B)** Protein-coding genes active in at least two cell lines were divided into three groups: unidirectional in all cell lines ‘Uni’, differently annotated ‘Mixed’, and bidirectional in all cell lines ‘Bi’.

The active genes were annotated as bidirectional based on either the presence of CAGE clusters on the opposite strand or the presence of a gene listed in the Ensembl database on the opposite strand within a short distance. The agreement between the two annotation methods was computed. On average, 86.5% of the active genes received the same annotation using both CAGE and Ensembl data (Table 1). This combined annotation was considered more certain and these genes were therefore the primary genes included in the subsequent analysis. To compare gene activity across cell lines, all protein-coding genes were subdivided based on the number of cell lines in which they were active (Figure 2A), revealing an enrichment of genes active in all or none of the cell lines. Since we considered several different CAGE RNA isolation conditions for some cell lines (see Table 1), we used the dataset resulting in the highest total number of active genes for this comparison. In total 95% of the genes active in at least two cell lines had the same directionality in all cell lines where they were active (Figure 2B).

To validate the annotations we compared the RNA Pol II and RNA-seq signals between the two groups of genes. For each gene we consider the upstream direction to be the 3′ to 5′ direction on the coding strand and the downstream direction to be the 5′ to 3′ direction. This definition is applicable also to genes defined as bidirectional, since each individual gene still has its coding sequence in only one direction of the TSS.

**Figure 3:**
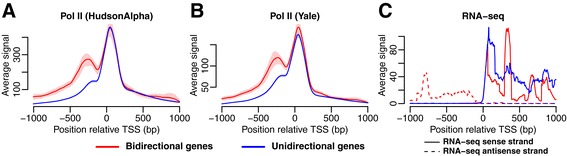
Gene annotations validated using RNA Pol II and RNA-seq signals. Results shown for K562 (cytosol, polyA-). **(A-B)** The average number of RNA Pol II reads (with 95% CI) in a region ±1 kb from the TSS based on **(A)** HudsonAlpha and **(B)** Yale ChIP-seq data. **(C)** Strand-specific RNA-seq signal. The sense strand (solid line) and antisense strand (dashed line) are shown separately.

As expected, the RNA Pol II-signal was higher upstream of the TSS in the bidirectional genes compared with the unidirectional, which agrees with higher antisense transcription in the bidirectional genes (Figure 3A-B). The presence of a weak RNA Pol II peak upstream of the TSS suggests that a small amount of divergent transcription may still take place for some genes in the unidirectional group, although the genes with the highest level of divergent transcription would have been generally detected as bidirectional using the CAGE data. Using strand-specific RNA-seq we confirmed that both bidirectional and unidirectional genes had RNA-seq signals downstream of the TSS, but that only the bidirectional genes had a signal upstream of the TSS (Figure 3C). These findings were consistent across all cell lines and CAGE RNA isolation conditions (Additional file 1: Figure S1, Additional file 1: Figure S2).

### Differences in HM and TF signals are related to the direction of transcription

**Figure 4:**
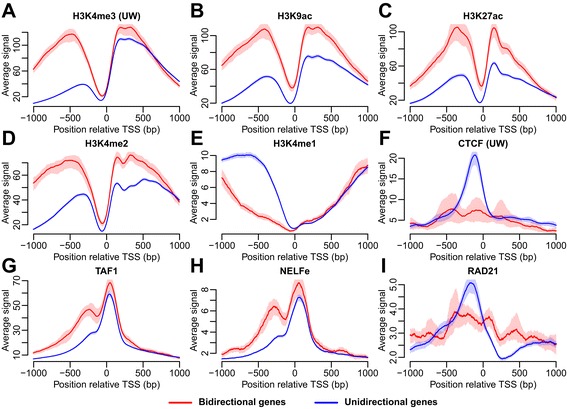
Differences in HM and TF signal between bi- and unidirectional genes. Results shown for K562 (cytosol, polyA-). The average signal (with 95% CI) is shown in a region ±1 kb from the TSS. The signal shown is either HMs typical for **(A** ***-*** **C)** promoters, **(D)** promoters and enhancers, **(E)** enhancers, or **(F** ***-*** **I)** TFs.

To investigate whether the HM signal upstream of the TSS was affected by the antisense transcription we computed the average HM signals for the bi- and unidirectional genes, respectively. We focused on genes annotated equally using both Ensembl and CAGE (Table 1). ENCODE HMs and histone variants from ‘Broad’ (H2A.Z, H3K27ac, H3K27me3, H3K36me3, H3K4me1, H3K4me2, H3K4me3, H3K9ac, H3K9me1, and H3K20me1) and ‘UW’ (H3K27me3, H3K36me3, H3K4me3) were included. The analysis was done for each combination of cell line and CAGE RNA isolation condition separately. The promoter marks H3K4me3, H3K9ac, and H3K27ac showed significant differences between the groups (Figure 4A-C). The signal upstream of the TSS was almost equally high as the downstream signal in the bidirectional genes whereas the upstream signal was significantly lower than the downstream in the unidirectional genes. The highest difference was observed for the H3K4me3 mark, which is known to be present around active promoters [1]. Furthermore, more than 91% of all Pol II regions correlate with H3K4me3 [1]. Our results show that most of the H3K4me3 signal upstream of the TSS derives from genes with antisense transcription (Figure 4A) suggesting that H3K4me3 is mainly deposited in sequences transcribed by RNA Pol II.

Additionally, we observed differences between the enhancer marks H3K4me1 and H3K4me2 (Figure 4D-E). H3K4me2 is a signal associated with both promoters and enhancers. Small differences in the H3K4me2 signal were observed in the same direction as for the promoter marks (Figure 4D). H3K4me1 had an opposite pattern (Figure 4E) with higher signal upstream of the TSS in the unidirectional genes. Since the monomethylation signal was one order of magnitude weaker than the H3K4me2/3 signal, we speculated that instead of being enriched in the unidirectional genes it was rather depleted in the bidirectional genes due to the bi- and trimethylation of the same residue. Furthermore, some promoters may act as enhancers [21] and it is possible that promoters of unidirectional genes have a higher tendency to act in this way.

Next we compared the TF binding between bi- and unidirectional genes including 83 ENCODE ChIP-seq datasets for TFs. The TF datasets are listed in (Additional file 2: Table S1). CTCF, TAF1, NELFe, and RAD21 showed significant differences between uni- and bidirectional genes (Figure 4F-I). The patterns for TAF1 and NELFe were very similar to the pattern for RNA Pol II with a higher peak upstream of the TSS in the bidirectional genes compared with the unidirectional ones (Figure 4G-H). A small tendency towards an upstream peak is still visible in the unidirectional genes, and it may be attributed to divergent transcription [8,9] that was not identified using the CAGE clusters.

TAF1 is a subunit of TFIID (transcription factor II D), which is one of the general TFs that constitute the RNA Pol II preinitiation complex. TAF1 is associated with active promoters and related to gene expression levels [22]. This association is driven by specific binding of the plant homeodomain (PHD) in the TFIID TAF3 subunit to H3K4me3-modified nucleosomes and enhanced by coinciding H3K9/14ac [23]. The pattern for TAF1 (Figure 4G) is therefore consistent with the observed differences in H3K4me3 and H3K9ac (Figure 4A-B).

NELFe (negative elongation factor E) is a part of the NELF complex that binds to RNA Pol II after initiation and causes pausing of Pol II elongation proximal to the promoter [24,25]. NELF has been shown to be present both downstream and upstream of the TSS, which indicates pausing in either direction [26]. We observed a higher NELFe signal upstream of the TSS of bidirectional genes (Figure 4H), corresponding to higher rate of antisense transcription.

Surprisingly, we also found a well-positioned CTCF peak centered 60-90 bp upstream of the TSS of the unidirectional genes (Figure 4F). This peak was observed in the unidirectional genes for all cell lines and CAGE RNA isolation conditions, but it was not present in the bidirectional genes. CTCF bound upstream of the TSS may thus act as a marker of unidirectional transcription. Since CTCF is known for creating boundaries, e.g. between enhancers and promoters or to act as a chromatin barrier [14] we hypothesized that the function of CTCF in the unidirectional genes may be to block the initiation of antisense transcription. Alternatively, CTCF may act by stalling the RNA Pol II [27,28] upstream of the TSS and thus increasing the likelihood that the antisense transcription is terminated. Increased CTCF binding (estimated via the motif) has previously been related to increased levels of H3K4me1 [6], which agrees with our observed differences in H3K4me1 between the two groups (Figure 4E).

Interestingly, this upstream CTCF peak in the unidirectional genes was observed both using the combined (CAGE and Ensemble) gene annotations (Figure 4F) and using only the CAGE-based annotations (Additional file 1: Figure S3F). However, using only the Ensembl-based annotations there was no significant difference between the two gene classes (Additional file 1: Figure S4F). This suggests that the CTCF binding is related to the initiation of transcription measured by CAGE rather than the gene organization measured using Ensembl.

To verify that the CTCF peak was not the result of a few outliers among the genes, we subdivided the ±1 kb window into 13 segments of length 153-154 bp. This particular subdivision was chosen to give the highest precision, without covering multiple nucleosomes in the same segment. We defined a CTCF peak to be at least a 100-fold enrichment of ChIP-seq signal over the background. The segment with the largest difference in prevalence of CTCF peaks covered the expected CTCF peak site (76-230 bp upstream of the TSS) and held a significant enrichment (p < 6.7·10^-7^, Fisher’s exact test) of CTCF peaks in the unidirectional genes compared with the bidirectional ones (Additional file 1: Figure S5E). The choice of enrichment threshold to define a CTCF peak may influence the results and several different thresholds (5, 10, 20, 50, 100, and 200-fold enrichment) were applied with similar results (Additional file 1: Figure S5).

Similarly to CTCF, we found a RAD21 peak upstream of the TSS in the unidirectional genes (Figure 4I). RAD21 is a cohesin subunit and has been shown to function together with CTCF [17]. In embryonic stem cells RAD21 is typically positioned at the 5′ end of the CTCF motif and 73% of the RAD21 binding sites have been found to overlap with CTCF [29].

We repeated the comparisons presented here using either annotations based solely on CAGE (Additional file 1: Figure S3) or Ensembl (Additional file 1: Figure S4). For the HMs and most TFs we observed no differences as compared with the combined analysis. However, for CTCF there was a small upstream peak also in the Ensembl-bidirectional group as discussed earlier (Additional file 1: Figure S4F), illustrating that the absence of antisense CAGE was the main feature that defined the upstream CTCF peak.

Results for all HMs and all TFs for all the tested cell lines and RNA isolation conditions studied are available in (Additional file 3: Figure S6) and (Additional file 4: Figure S7). We made interesting observations on the H2A.Z histone variant. H2A.Z is enriched mainly upstream of the TSS in human [1], both upstream and downstream in mouse and yeast [30,31], but mainly downstream of the TSS in *Drosophila* [32] and *Arabidopsis* [33]. In unidirectional genes we found that H2A.Z showed strongest signal upstream of the TSS but in bidirectional genes the signal was stronger downstream of the TSS (Additional file 3: Figure S6 A1-I1). Thus, high levels of H2A.Z downstream of the TSS may be indicative of antisense transcription in human, but since the positioning of H2A.Z differs in other species it may not be a causative relation.

### Alternative TSSs do not affect the signal upstream of the TSS

We speculated that the peak upstream of the TSS, which was observed for several HMs, could be influenced by genes with an upstream alternative TSSs (Figure 1C). Within the previously annotated groups of unidirectional genes we identified genes without any upstream CAGE clusters on the same strand. Then the HM and TF signals were compared between this subgroup of unidirectional genes without upstream alternative TSSs and all unidirectional genes (Additional file 1: Figure S8). Noticing only very small differences between these groups, we concluded that the occurrence of upstream TSSs did not significantly affect the analysis.

### Uni- and bidirectional genes are transcribed at similar levels

Next, we speculated that the observed differences in HM and TF signal between the bidirectional and unidirectional genes might be associated with overall differences in transcription between the two groups. To test this idea, we subdivided the genes into four equally sized transcription level bins (‘Lowest’, ‘Mid-low’, ‘Mid-high’, and ‘Highest’) prior to the gene annotation, and computed the signal for each bin separately. Had the observed differences been related to overall expression differences, then we would have expected e.g. bidirectional genes to preferentially fall into the highest expressed bin and unidirectional genes into the lowest expressed bin. However, the distribution of genes was nearly uniform across the expression bins for all cell lines (Additional file 1: Table S2). Computing the RNA Pol II and RNA-seq signal for the transcription level bins confirmed that the ‘Highest’ bin also had the highest Pol II and RNA-seq signal (Additional file 1: Figure S9).

**Figure 5:**
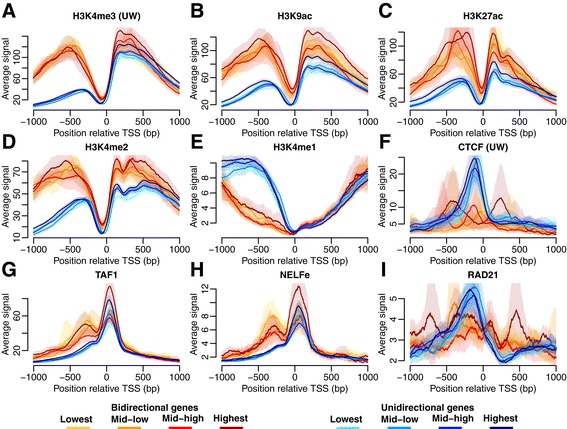
Differences between bi- and unidirectional genes subdivided into four gene expression bins. Results shown for K562 (cytosol, polyA-). The average HM and TF signal (with 95% CI) is shown in a region ±1 kb from the TSS. The signal shown is either HMs typical for **(A** ***-*** **C)** promoters, **(D)** promoters and enhancers, **(E)** enhancers, or **(F** ***-*** **I)** TFs.

To test if the identified differences in HM and TF signals between bi- and unidirectional genes were associated with the gene expression levels, we reanalyzed the HMs and TFs with the genes subdivided into bins according to their transcription level (Figure 5). Although there are small variations in the signal, all qualitative results were preserved. Notably, the well-positioned CTCF and RAD21 peaks were clearly present in all transcription level bins of the unidirectional genes, whereas they were missing in all bidirectional bins.

### HM and TF signals are related to the level of antisense transcription

**Figure 6:**
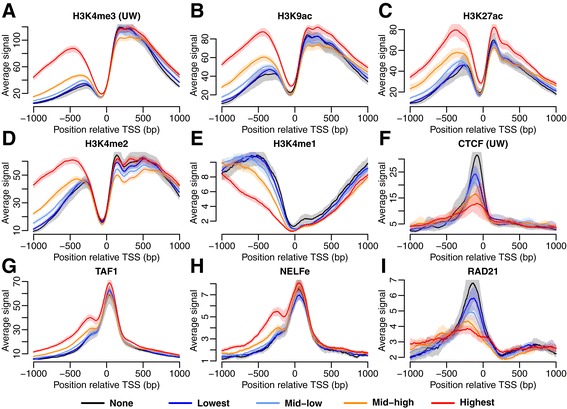
Differences in HM and TF signal for genes grouped by the level of antisense transcription. Results shown for K562 (cytosol, polyA-). The average signal (with 95% CI) is shown in a region ±1 kb from the TSS. The signal shown is either HMs typical for **(A** ***-*** **C)** promoters, **(D)** promoters and enhancers, **(E)** enhancers, or **(F** ***-*** **I)** TFs.

To verify that the choice of thresholds used to divide the active genes into the bi- and unidirectional groups did not affect the results, we reanalyzed the data in a threshold-independent way. In each cell line, all active protein-coding genes were subdivided into five groups based on level of antisense transcription (‘None’, ‘Lowest’, ‘Mid-low’, ‘Mid-high’, and ‘Highest’). The same HMs and TFs as earlier were analyzed confirming the previous observations (Figure 6). Notably, the HM and TF signals were related to the level of antisense transcription, represented by the different bins. For instance, the height of the upstream CTCF peak was negatively associated with the level of antisense transcription (Figure 6F). Subsequently, the highest CTCF peak was observed for the ‘None’ group without any antisense CAGE-tags, a slightly lower peak was observed for the ‘Lowest’ group, and so on.

Since there is still a small peak upstream of the TSS, e.g. for H3K4me3 in the ‘None’ group, this association between the level of antisense transcription for a bin and its signal strength is important in illustrating that most of the upstream signal may indeed be explained by the antisense transcription. The part of the signal that is not explained may be due to small levels of divergent transcription that are not detectable by CAGE.

### CTCF is negatively correlated to H3K4me3, H3K9ac, and H3K27ac in genes with an upstream CTCF peak

**Figure 7:**
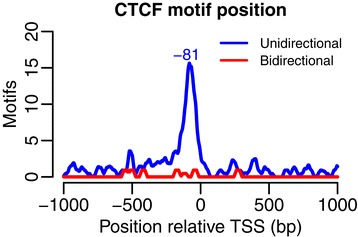
Position of the CTCF motif in K562 (cytosol, polyA-). The per-bp motif coverage was computed in a region ±1 kb from the TSS for uni- and bidirectional genes separately. The signal shown was averaged over a ±20 bp window and the position with the highest motif enrichment marked.

To further study CTCF we compared the CTCF motif distribution between uni- and bidirectional genes. Motifs within 1 kb from the TSSs were identified. An enrichment of motifs was found for the unidirectional genes in K562 with a peak 81 bp upstream of the TSS (Figure 7), but no enrichment was observed in the bidirectional genes. Similar patterns were found for each individual cell line (Additional file 1: Figure S10).

**Figure 8:**
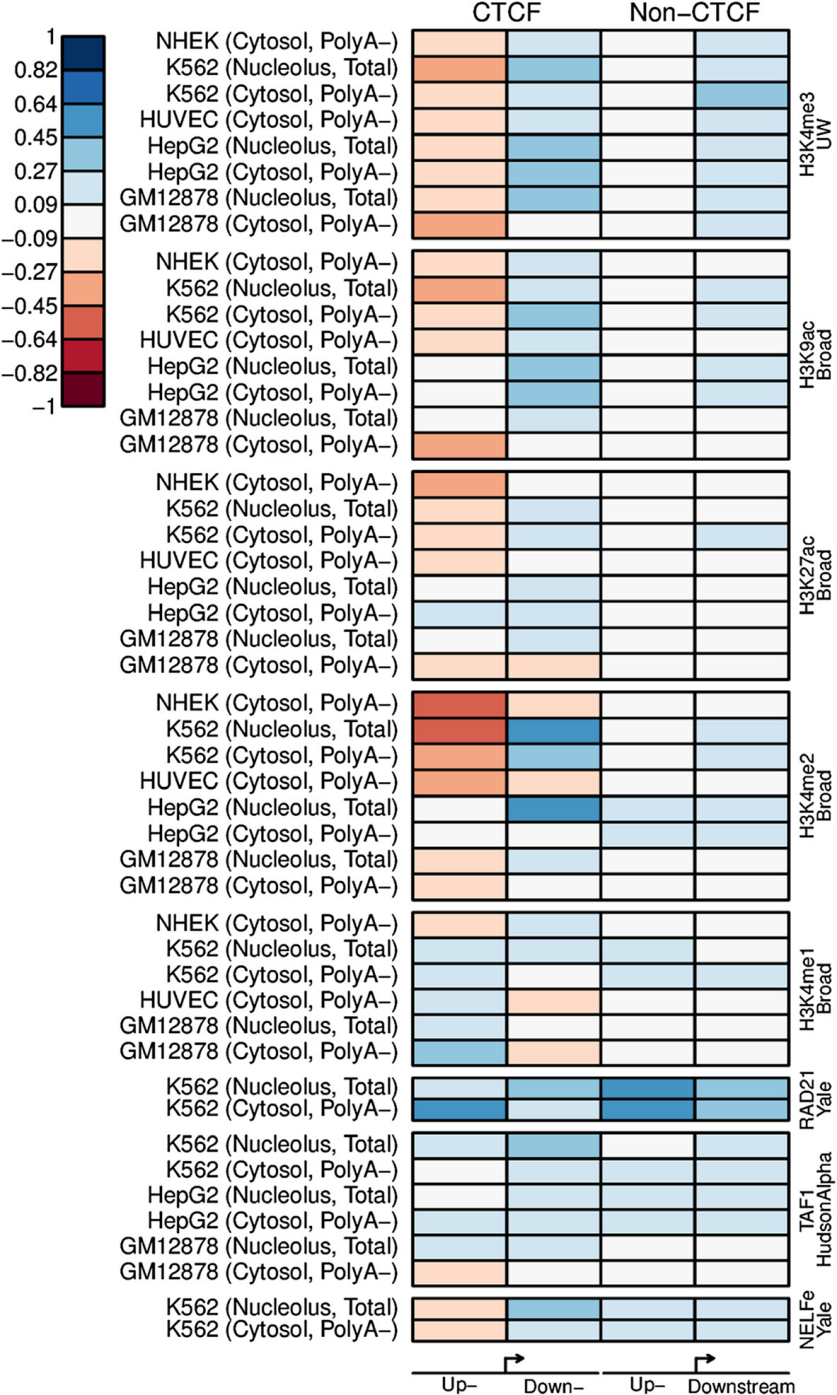
Correlation between the CTCF signal and the HM or TF signal. The results are shown for several cell lines annotated using CAGE from different subcellular locations (indicated by the left column). The genes were subdivided into genes with a well-positioned CTCF peak upstream of the TSS and those without (‘CTCF’ and ‘Non-CTCF’, respectively). The CTCF signal was computed for the whole region ±1 kb from the TSS, whereas the HM and TF signals (name and ENCODE lab specified to the right) were subdivided into the signal upstream and downstream of the TSS. The correlation between the total CTCF signal and the HM or TF signal up- and downstream of the TSS is shown on a scale from dark red (high negative correlation), to white (no correlation), to dark blue (high positive correlation).

Using the identified motif site, we selected unidirectional genes that had both a CTCF motif close to the motif peak and a ChIP-seq peak supported by at least 100 reads. These genes were defined as the ‘CTCF’ group of genes and the rest of the unidirectional genes were defined as the ‘Non-CTCF’ group. For both groups we computed the correlation between the CTCF signal in a ±1 kb window from the TSS and the HM and TF signal upstream or downstream of the TSS, respectively. The results (Figure 8) showed that the CTCF level was negatively correlated to the upstream signal of H3K4me3, H3K9ac, and H3K27ac in the ‘CTCF’ group, whereas the signals were uncorrelated in the ‘non-CTCF’ group. Conversely, the downstream signals of H3K4me3, H3K9ac, and H3K27ac were positively correlated to the CTCF signal, with a higher correlation in the CTCF genes. RAD21 was positively correlated to CTCF in both groups of genes and both upstream and downstream of the TSS, illustrating a strong co-occurrence of RAD21 and CTCF.

### CTCF co-occurs with cohesin and is associated with unidirectional transcription

**Figure 9:**
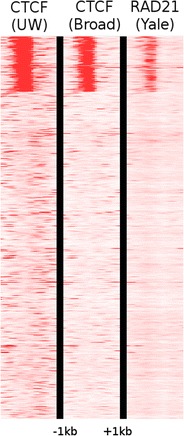
Co-occurrence of CTCF and RAD21. Active genes in K562 (cytosol, polyA-) were clustered into two clusters based on CTCF and RAD21 signal within 1 kb from the TSS.

To illustrate the co-occurrence of CTCF and cohesin we clustered all active protein-coding genes in K562 into two clusters using CTCF and RAD21 ChIP-seq data. The first cluster held 652 genes with co-occurring CTCF and RAD21 signal, whereas the second cluster held 3848 genes without the co-occurring signals (Figure 9). The CTCF and RAD21 cluster represented 14.7% of the active genes including 15.5% of the unidirectional genes but only 7.4% of the bidirectional genes, demonstrating that co-occurring CTCF and RAD21 was significantly associated with unidirectional genes (p = 1.2·10^-5^, Fisher’s exact test).

## Discussion

Several post-translational HMs are associated with gene activation or repression but the mechanisms are not fully understood yet. If this association is causal, the HMs may either be deposited there first to regulate the transcription or, alternatively, deposited as a consequence of the gene being transcribed. Different mechanisms have been discussed in the past and correlation has often been interpreted as causality [4]. However, since no sequence specificity has been observed for the histone-modifying enzymes, other mechanisms must be involved in identifying genes to promote or repress transcription. Another option is that both gene transcription and HMs are a result of the action of sequence specific TFs. In this model, the HMs, once established, could function as a cellular memory in a more complex regulatory machinery, but would not be the underlying cause of transcription themselves. Nevertheless, HMs are often referred to as ‘activating’ and ‘repressing’, implying a causality [4].

The study of how TFs and HMs affect gene regulation is complicated by the presence of antisense transcription. For instance, H3K4me3 has been reported to be located around the promoter of active genes [1]. In our study, however, we observed that the H3K4me3 signal upstream of the TSS mainly appeared in bidirectional genes, suggesting that it does not mark a region around transcriptional initiation as previously reported, but rather the transcribed sequences.

Here, we identified bi- and unidirectional genes and compared them with respect to HMs and TFs in the promoter region. We found that the promoter marks H3K4me3, H3K9ac, and H3K27ac had higher signal upstream of the TSS in bidirectional genes compared with unidirectional. A similar observation was made for the promoter/enhancer mark H3K4me2. These differences in HM enrichment were consistent in six different cell lines using CAGE data from several RNA isolation conditions. We showed that the HM differences were not linked to differences in gene expression between bi- and unidirectional genes and that the differences increased with higher levels of antisense transcription. These findings agree with a previous observation of weaker H3K4me2 and H3ac signals upstream of the TSS in genes without a significant divergent transcription [9,26] measured in IMR90 cells using GRO-Seq. Unfortunately, we did not find any published GRO-seq data for the cell lines we studied to compare our results with.

Had the studied HMs occurred outside of the actually transcribed region their gene-regulatory role would have been supported since it would have suggested that a process separate from the transcription must add the HMs. By contrast, we observed differences in the HM signal upstream of the TSS between bi- and unidirectional genes, suggesting that the transcription either causes the HMs to be deployed there, or that they both have a common cause. Furthermore, roughly equal levels of sense and antisense RNA have been observed for a majority of active promoters using GRO-seq [9]. This would suggest that genes with similar levels of HM marks both upstream and downstream of the TSS could be expected. Indeed, the HMs both upstream and downstream of the TSS had similar enrichment in the group of genes that were defined as bidirectional.

Additionally, analysis of TF occupancy in the two groups of genes revealed some TFs with interesting differences in enrichment. TAF1 and NELFe had ChIP-seq signals similar to that of RNA Pol II with higher signal upstream of the TSS of the bidirectional genes compared with the unidirectional ones. Both TFs are tightly coupled to the transcriptional initiation. TAF1 is a subpart of TFIID, which is part of the Pol II preinitiation complex [22]. NELFe binds to Pol II and is involved in pausing of the initial elongation [24]. The TFIID subunit TAF3 has been shown to bind specifically to H3K4me3-modified nucleosomes that is also enhanced by coinciding H3K9/14ac [23].

Interestingly, CTCF and RAD21 were found to have a well-positioned peak approximately 60-80 bp upstream of the TSS in the unidirectional genes but not in the bidirectional ones. The peak height was associated with the level of antisense transcription. Since CTCF is known for creating boundaries between different regions [14], we speculated that the function here might be to block the initiation of antisense transcription. Cohesin forms circular structures around the DNA molecule e.g. keeping two sister chromatids together. The cohesin ring has been shown to be mobile and it has been suggested that cohesin is pushed away by the transcription complex [34], which would cause it to be depleted upstream of the TSS of bidirectionally transcribed genes. However, since CTCF was strongly co-localized with cohesin upstream of the TSS of unidirectional genes, CTCF may be involved in the positioning of cohesin instead. CTCF and cohesin have been shown to stall the RNA Pol II [28,35] and transcription in the antisense direction may be more likely to terminate due to RNA Pol II stalling, which could be the mechanism linking these two factors to the direction of transcription.

We have previously analyzed H3K4me3 and using k-means clustering verified the existence of several subgroups of promoters with distinct signals that differ significantly from the average of all genes [36]. In the present study we subdivided the actively transcribed genes into uni- and bidirectional genes and found that the HM signals highly differed between the groups, observing promoter-associated modifications located in essence in transcribed sequences. Had the HMs located there been the main force to decide transcription level there would have had to exist a specific process to place the HMs on these nucleosomes prior to transcription. The molecular details of such a putative mechanism are far from clear. Another alternative is that the enzymes adding the promoter-associated HMs are part of the RNA Pol II complex. The main force to regulate gene activity would then be the binding of TFs, which is consistent with ENCODE data [20]. Nevertheless, the HMs are important by creating a memory in chromatin making it easier for new rounds of transcription to occur [23,26].

## Conclusions

The HMs H3K4me3, H3K9ac, H3K27ac, and H3K4me2 were identified to be more enriched upstream of the TSS in bidirectional genes compared with unidirectional genes. These observations are compatible with promoter-specific HMs being deposited as a consequence of transcription, although a deeper understanding of the biological mechanisms is still needed. Furthermore, binding of the TFs NELFe and TAF1 were shown to be related to the RNA Pol II signal, which differed between bi- and unidirectional genes, and a potentially new role of CTCF and cohesin in regulating the direction of transcription was found.

## Methods

### Cap analysis of gene expression data

CAGE clusters and aligned reads for the human genome (NCBI36, hg18) produced by the RIKEN lab were downloaded from the ENCODE repository [20] at UCSC. The CAGE RNA had been isolated from different subcellular locations, using different RNA extracts (polyA+, polyA-, or total RNA). We included three of the isolation conditions in this study: 1) polyA- from the cytosol (measured for GM12878, HepG2, HUVEC, K562, and NHEK), 2) total RNA from the nucleolus (GM12878, HepG2, and K562), and 3) polyA- from whole cells (H1hESC). The first isolation condition was selected since it covered the highest number of cell lines, the second was selected to also cover polyA+ RNA, and the third was selected to include the H1hESC cell line in the study. Most of the downloaded datasets contained several million clusters. To select the clusters that were most likely to correspond to real promoters, a threshold on the cluster score was defined for each dataset. This threshold was set to select at most 29,857 clusters in each sample, which is the number of promoters previously identified using CAGE for THP-1 myelomonocytic leukemia cells in an extensive study [37].

### Identification of bi- and unidirectional genes

Annotation of genes as bi- or unidirectional was done separately for each cell line. We started from all 19,950 protein-coding genes in the Ensembl (*H. sapiens* 54_36p) database [18] and excluded those that were not active in the selected cell line. A gene was considered active if it had at least one CAGE cluster on the same strand and within 10 bp from the TSS. In the comparison of gene activity between the cell lines (Figure 2A), the CAGE samples which gave the highest number of active genes were used for each cell line.

Next, we used two different approaches to identify bidirectional genes among the active genes. Firstly, a gene was defined as bidirectional if there was another gene annotated in Ensembl with a TSS on the opposite strand within 1 kb from the TSS. Secondly, a gene was considered bidirectional if there was a CAGE cluster on the opposite strand within 1 kb from the TSS. For each approach, a gene was considered unidirectional if it was active and not identified as bidirectional. Finally, the annotations using either Ensembl or CAGE were compared and only genes annotated similarly using both methods were included in this study.

### Analysis of ChIP-seq data for HMs, TFs, and Pol II

Aligned ChIP-seq reads for the human genome from the Broad, HudsonAlpha, UW, and Yale labs were downloaded from the ENCODE repository [20] at UCSC. All datasets were aligned to the NCBI36/hg18 assembly of the human genome, except H2A.Z, which was aligned to GRCh37/hg19. In this study we included HMs, TFs, and the RNA Pol II occupancy using 13 datasets describing different HMs and histone variants, 2 describing RNA Pol II, and 83 describing different TFs. These datasets are listed in (Additional file 2: Table S1). Several labs have contributed with data for some targets, and the lab name is provided in this manuscript whenever the source of the data is ambiguous.

The biological replicates were merged and the reads were processed using the SICTIN [38] tool *build_binary* into a binary format in which the number of reads at each genomic position is explicitly saved. Since the read length may differ between different labs, the reads were extended to 147 bp which is the approximate size of a nucleosome. Footprints of the average number of mapped reads in a region of ±1 kb around the TSSs for groups of genes were constructed using SICTIN *make_footprint* [38]. Genomic coordinates in hg18 format were converted to hg19 using liftOver [39] before retrieving the footprint signal for H2A.Z. The 95% confidence interval of the mean was estimated with bootstrapping, resampling the genes 100 times.

### Analysis of RNA-seq data

Single strand-specific aligned RNA-seq data for the human genome (NCBI36, hg18) from the Caltech lab was downloaded from the ENCODE repository [20] at UCSC (GEO accession GSE23316). Reads from biological replicates of the same cell line were merged and the data was converted from bed12 to bed with gapped reads split into multiple non-gapped rows. The SICTIN tool *build_binary* was used to convert the aligned reads into a binary format. Footprints were constructed using SICTIN *make_footprint* for each strand separately.

### Computation of CTCF peaks in individual genes

The studied region of ±1 kb from the TSS was subdivided into 13 segments of length 153-154 bp. For each gene from the unidirectional or bidirectional group the highest number of overlapping CTCF reads was computed within each of the 13 segments. The number of overlapping reads was compared with the genomic average to measure the per-segment enrichment of reads. A segment was considered to contain a peak if it had a certain enrichment of ChIP-seq reads compared with the background. Different such thresholds for the enrichments were applied, including a 5, 10, 20, 50, 100, or 200-fold enrichment. The percentage of genes with a CTCF peak was computed for each segment for the unidirectional and the bidirectional genes separately. Fisher’s exact test was applied to test if the difference between the two groups was significant (p < 0.05). In total, considering 13 intervals and six thresholds 78 significance tests were performed and Bonferroni correction was applied to correct the p-values for multiple testing.

### Identification of genes with alternative TSSs

For each cell line the unidirectional genes were additionally filtered with respect to CAGE clusters within 1 kb upstream on the same strand as the TSS. These genes were defined as ‘unidirectional genes without upstream TSS’ and were compared with the group of ‘bidirectional’ and ‘unidirectional’ genes.

### Analysis of the impact of transcriptional level

For each cell line the active genes were subdivided into four approximately equally sized transcription level bins (‘Lowest’, ‘Mid-low’, ‘Mid-high’, and ‘Highest’) determined by the number of raw CAGE tags on the same strand and within 10 bp from the TSS. The annotation of genes as bi- or unidirectional was then redone on each of these four groups as described earlier. The distribution of gene annotations across the transcription level bins was computed. The HM, TF, Pol II, and RNA-seq signals were computed for each subgroup of genes separately.

### Assessing the impact of the antisense transcription level

All active genes were subdivided into five groups based on the number of raw CAGE tags within 1 kb from the TSS on the opposite strand. Since most cell lines and isolation conditions had a large number of genes without any opposite CAGE tag, these genes were put into an own group (‘None’) for genes without CAGE evidence of antisense transcription. The remaining genes were subdivided into four equally sized CAGE-tag bins (‘Lowest’, ‘Mid-low’, ‘Mid-high’, and ‘Highest’), representing different levels of antisense transcription. The HM and TF signals were computed for each subgroup of genes separately.

### Identification of CTCF motifs

The CTCF motif was downloaded from the JASPAR database [40]. FIMO version 4.9.1 [41] was used to search for the motif within 1 kb from the TSS using a q-value threshold of 0.05 and otherwise default parameters. To avoid that differences in the number of bi- and unidirectional genes affected the reporting thresholds, FIMO was run once per cell line and CAGE RNA isolation condition for all active protein-coding genes. The identified motifs were then mapped to the groups of bi- and unidirectional genes according to their genomic coordinates.

The CTCF motif coverage was computed for each cell line in a ±1 kb region from the TSS using the earlier identified motif positions. The signal was smoothened by computing a ±20 bp average in each position, and the position with the highest motif signal was identified as the motif site.

### Identification of genes with a well-positioned CTCF peak

For each cell line and CAGE RNA isolation condition, unidirectional genes with a CTCF motif within 100 bp from the motif site and with at least 100 ChIP-seq reads for CTCF (ENCODE, UW) were defined as genes with a well-positioned CTCF peak (the ‘CTCF’ group). The remainder of the unidirectional genes was defined as the ‘Non-CTCF’ group. For each group, the correlation was computed between the total CTCF signal and the HM or TF signal up- or downstream of the TSS, respectively.

### Computing co-occurrence of CTCF and RAD21

All genes that were active in K562 (using CAGE from cytosol, polyA-) were clustered with respect to their CTCF (using both UW and Broad) and their RAD21 signal (Yale). The clustering was performed using seqMINER [42] with kMeans linear clustering, two clusters, and considering a 1 kb window from the TSS. The association between cluster and directionality of the genes was verified using Fisher’s exact test.

## Additional files

Additional file 1:
**Figure S1-Figure S5,** **Figure S8-Figure S10,** **and** **Table S2.**
Additional file 2: Table S1. ChIP-seq datasets. Additional file 3: Figure S6. Differences in HM signals between bi- and unidirectional genes annotated using both Ensembl and CAGE shown for all cell lines and 13 HM and histone variant datasets. The average signal (with 95% CI) is shown in a region ±1 kb from the TSS. Additional file 4: Figure S7. Differences in TF signals between bi- and unidirectional genes annotated using both Ensembl and CAGE shown for all cell lines and 83 TF datasets. The average signal (with 95% CI) is shown in a region ±1 kb from the TSS.

## Abbreviations

1 kb: 1000 bp
CAGE: Cap analysis of gene expression
CTCF: CCCTC-binding factor
H: Histone
HM: Histone modification
NELFe: Negative elongation factor E
PHD: Plant homeodomain
SNP: Single nucleotide polymorphism
TF: Transcription factor
TFIID: Transcription factor II D
TSS: Transcription start site
