# Supplementary material for: Different distribution of histone modifications in genes with unidirectional and bidirectional transcription and a role of CTCF and cohesin in directing transcription
Source: BMC Genomics. 2015 Apr 15;16(1):300. doi: 10.1186/s12864-015-1485-5 (PMC4446127; doi:10.1186/s12864-015-1485-5)
Supplement: Additional file 1: — Figure S1-Figure S5, Figure S8-Figure S10, and Table S2. [file 12864_2015_1485_MOESM1_ESM.doc]

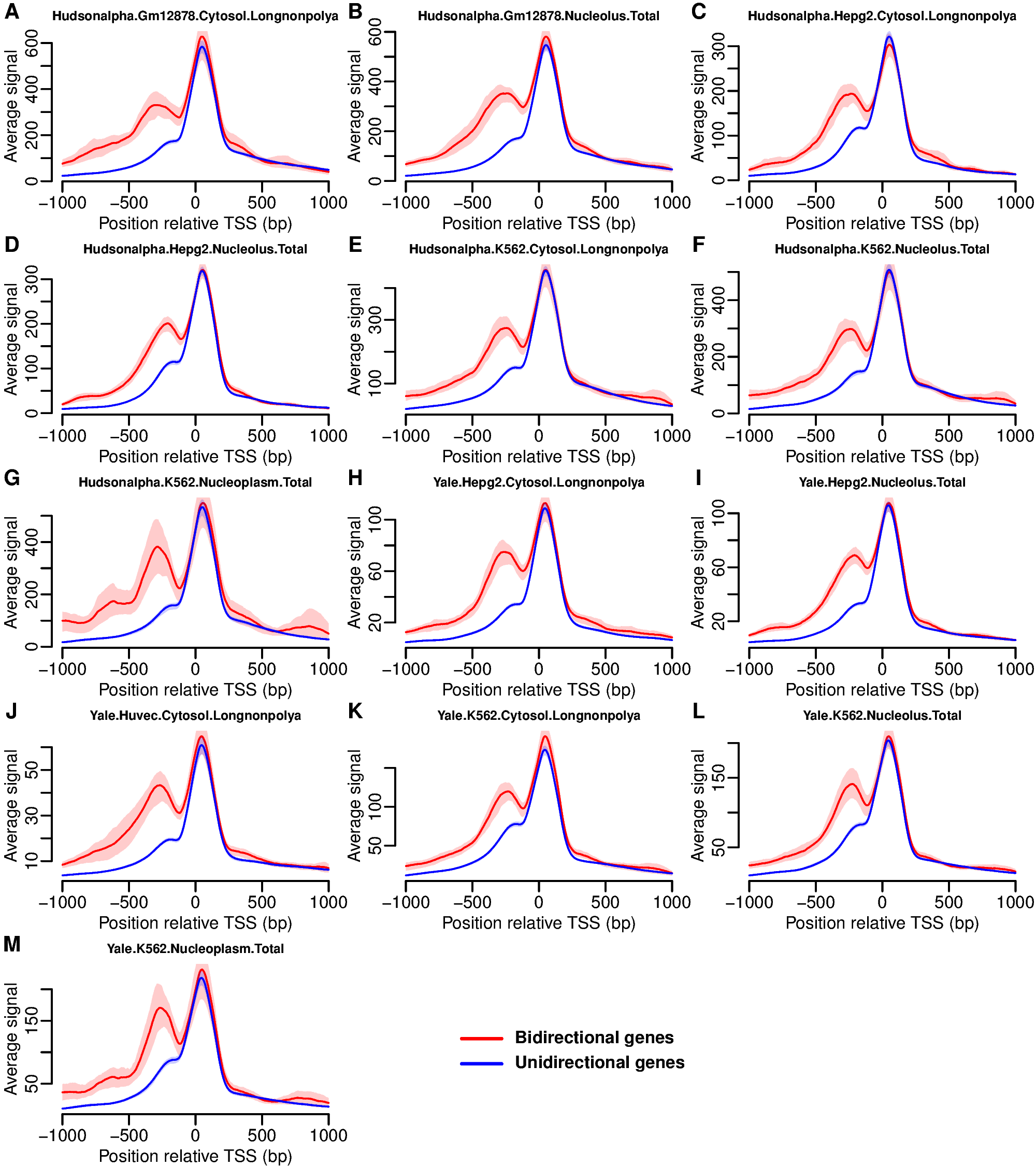


**Figure S1.** Gene annotations for all cell lines validated using RNA Pol II. The average number of RNA Pol II reads (with 95% CI) in a region ±1 kb from the TSS.


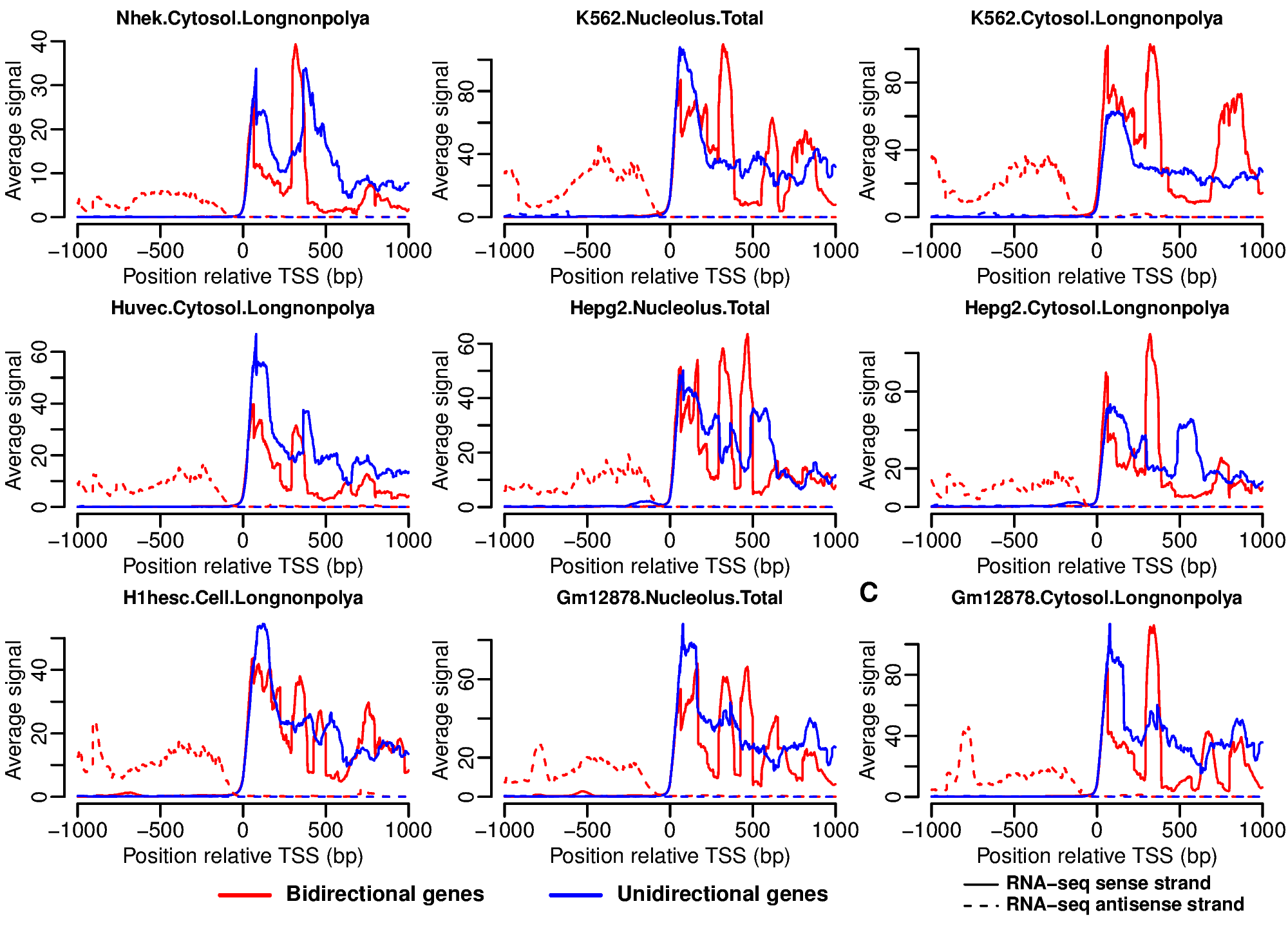


**Figure S2.** Gene annotations for all cell lines validated using strand-specific RNA-seq. The average number of RNA-seq reads shown for the sense strand (solid line) and antisense strand (dashed line) separately.


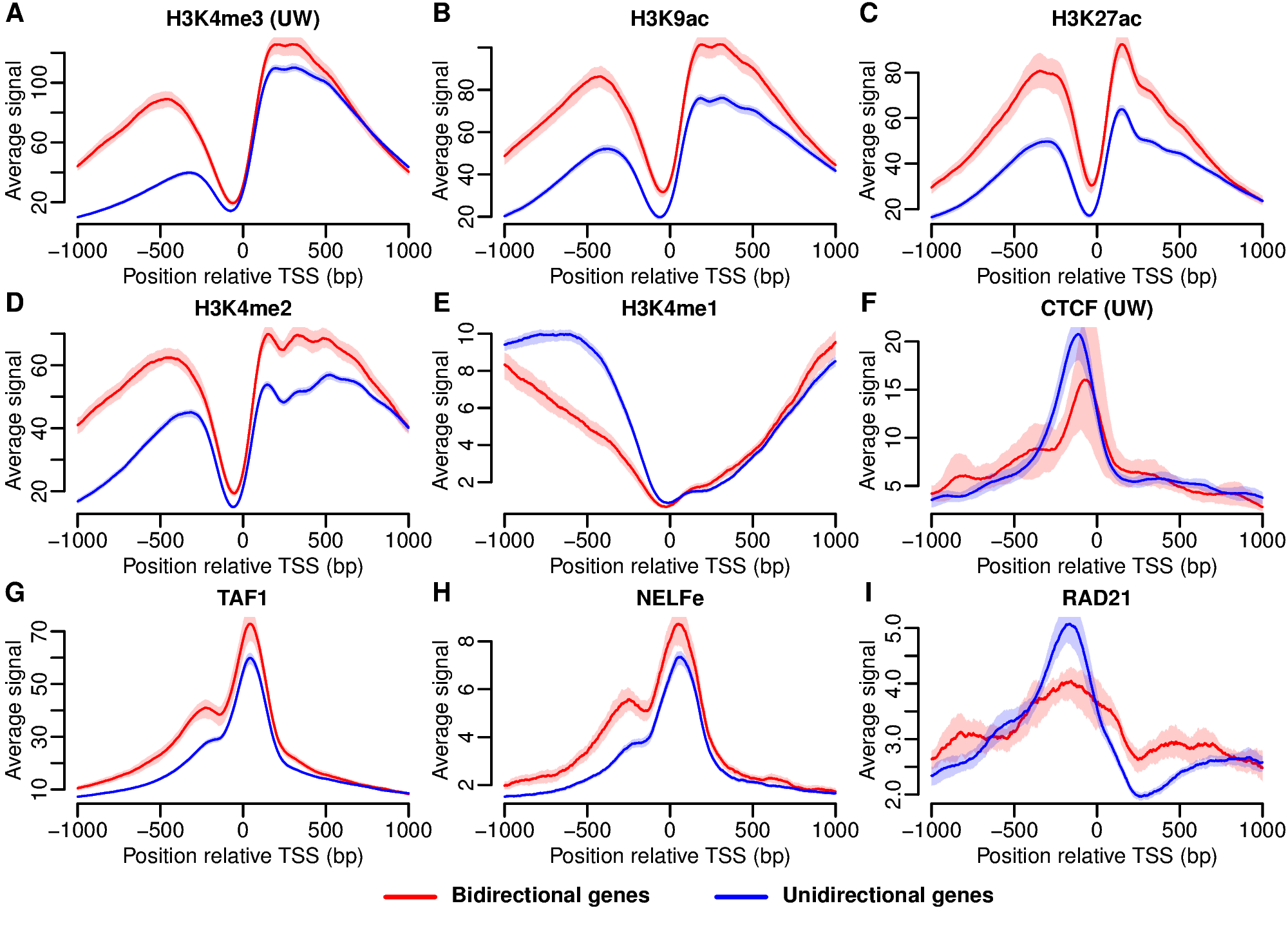


**Figure S3.** Differences in HM and TF signal between bi- and unidirectional genes annotated using Ensembl shown for K562 (cytosol, polyA-). The average signal (with 95% CI) is shown in a region ±1 kb from the TSS. The signal shown is either HMs typical for (**a***-***c**) promoters, (**d**) promoters and enhancers, (**e**) enhancers, or (**f***-***i**) TFs.


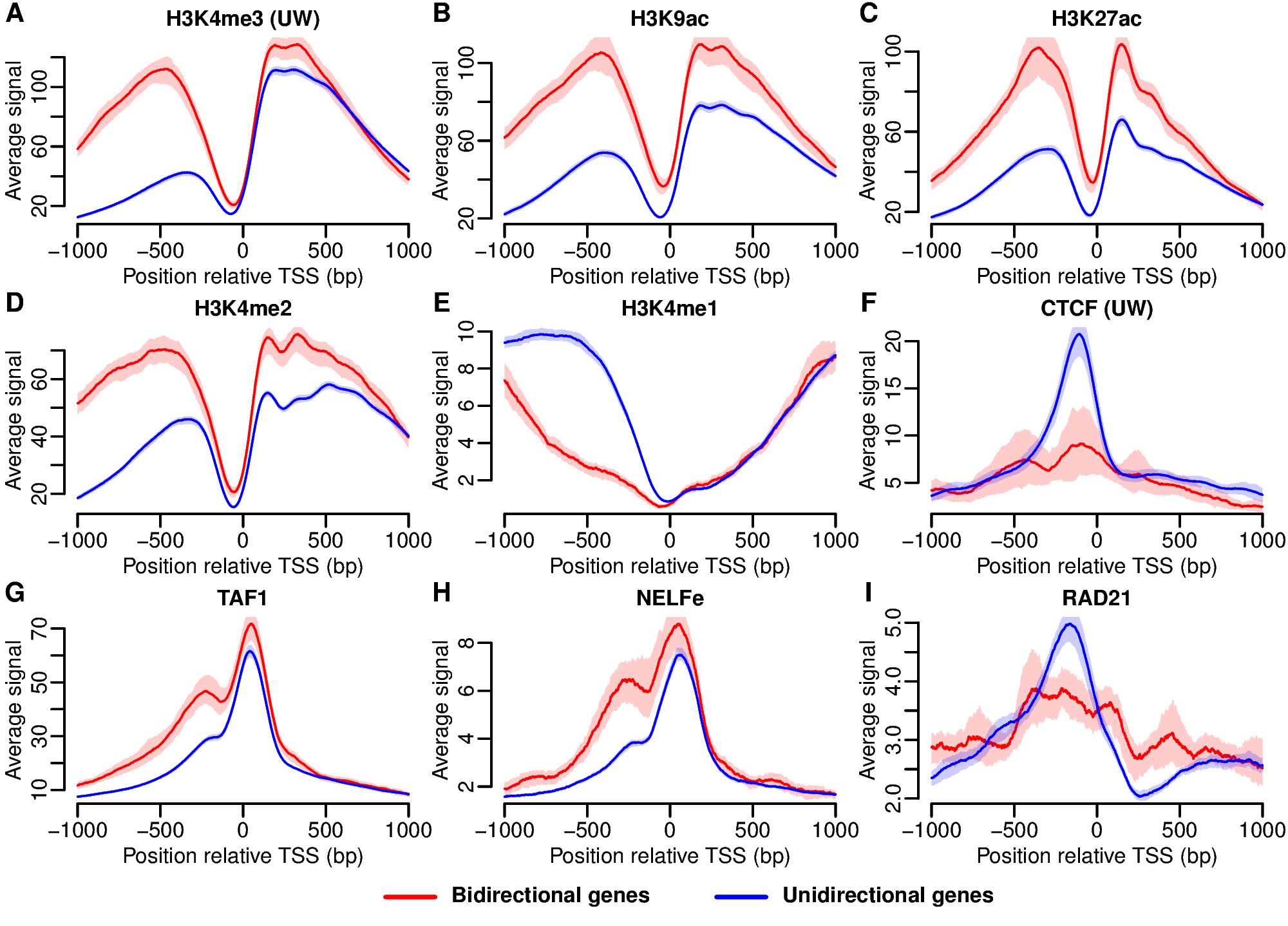


**Figure S4.** Differences in HM and TF signal between bi- and unidirectional genes annotated using CAGE shown for K562 (cytosol, polyA-). The average signal (with 95% CI) is shown in a region ±1 kb from the TSS. The signal shown is either HMs typical for (**a***-***c**) promoters, (**d**) promoters and enhancers, (**e**) enhancers, or (**f***-***i**) TFs.

**
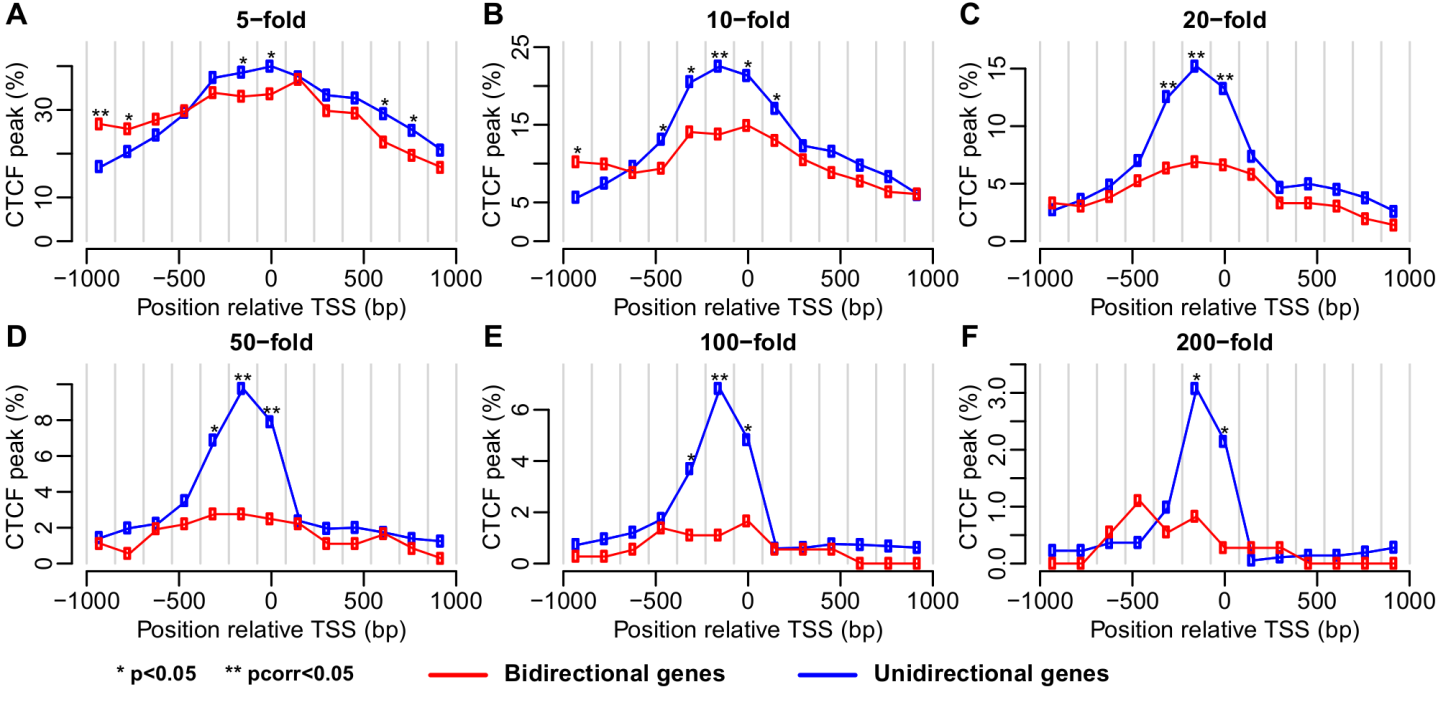
**

**Figure S5.** Results shown for K562 (cytosol, polyA-). Prevalence of CTCF peaks with signal at least (**a**) 5, (**b**) 10, (**c**) 20, (**d**) 50, (**e**) 100, or (**f**) 200-fold enriched over the average signal in 13 segments. The fraction of genes with a CTCF peak shown for bi- and unidirectional gens separately. In each segment, the ‘*’ marks a significant difference (*p*<0.05, Fisher’s exact test) in the number of peaks between the two groups, and the ‘**’ marks a significant difference after Bonferroni correction.


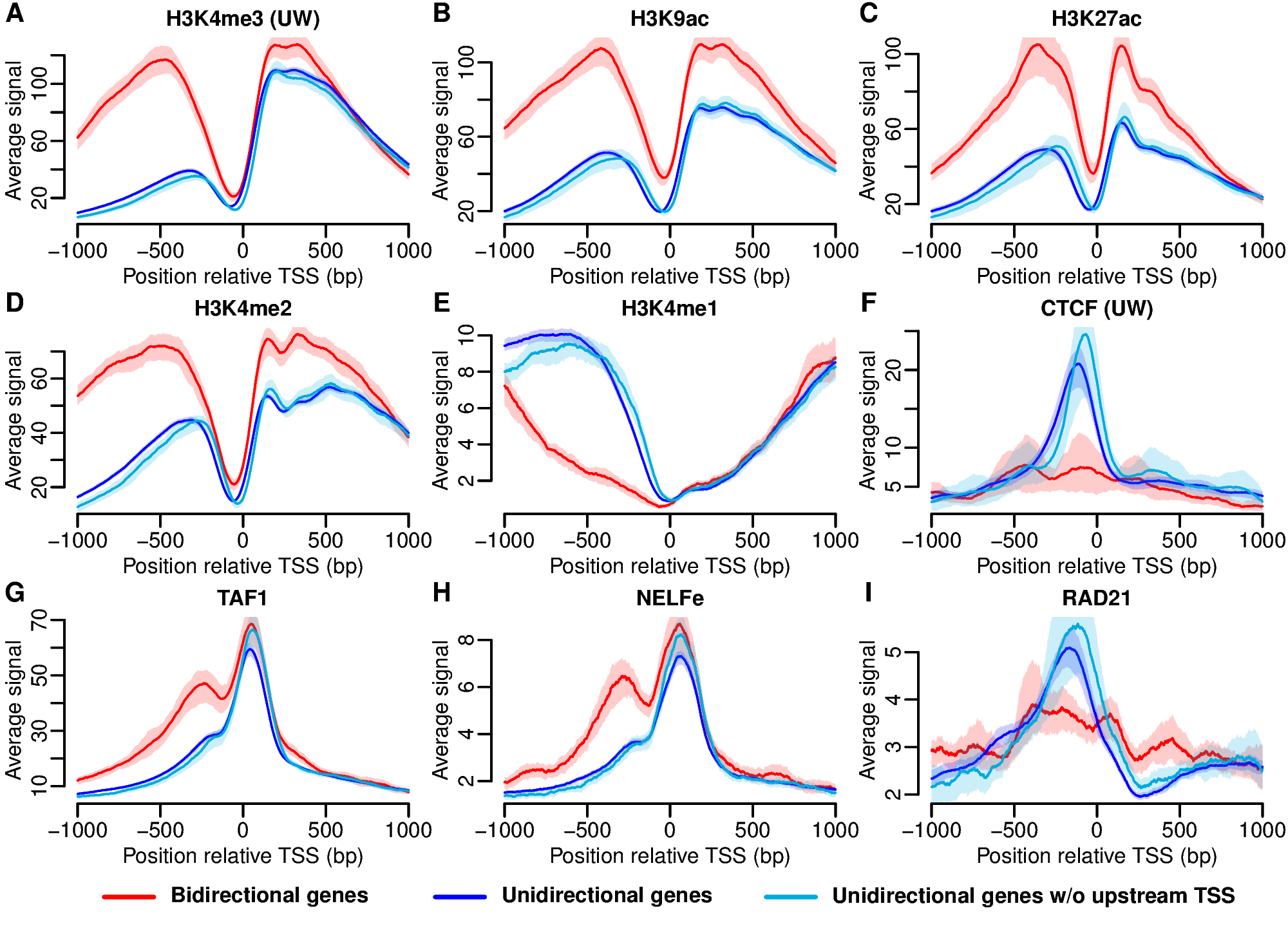


**Figure S8.** Differences in HM and TF signal between bidirectional, unidirectional, and unidirectional genes without any upstream TSS shown for K562 (cytosol, polyA-). The average signal (with 95% CI) is shown in a region ±1 kb from the TSS. The signal shown is either HMs typical for (**a***-***c**) promoters, (**d**) promoters and enhancers, (**e**) enhancers, or (**f***-***i**) TFs.


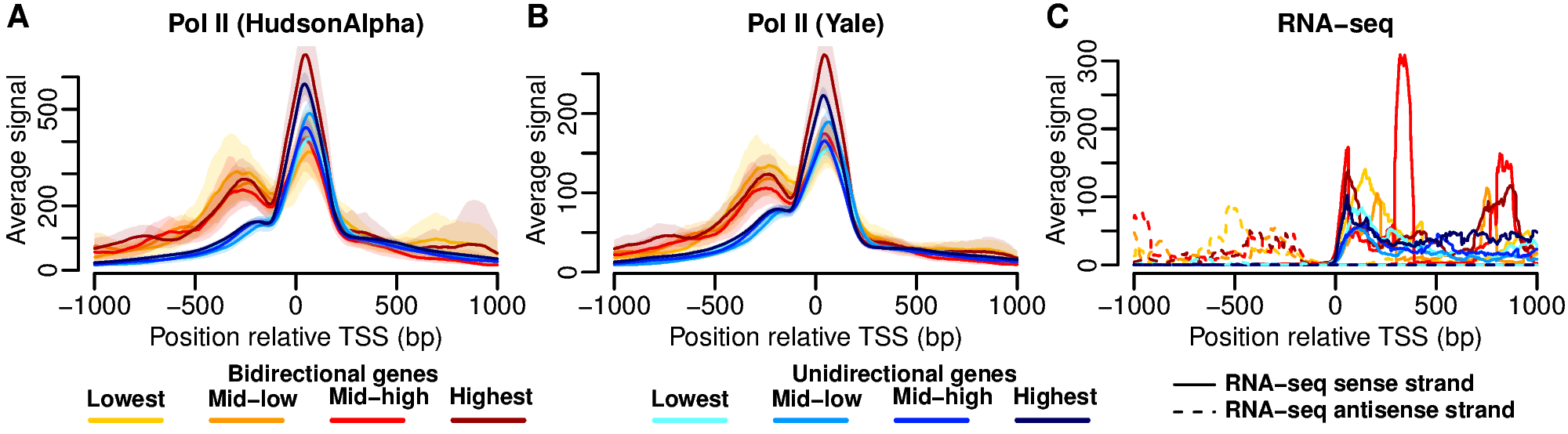


**Figure S9.** Gene annotations for K562 (cytosol, polyA-) validated using RNA Pol II and RNA-seq signals. Each group of genes was divided into four expression bins based on CAGE. (**a**-**b**) The average number of RNA Pol II reads (with 95% CI) in a region ±1 kb from the TSS based on (**a**) HudsonAlpha and (**b**) Yale ChIP-seq data. (**c**) Strand-specific RNA-seq signal. The sense strand (solid line) and antisense strand (dashed line) are shown separately.


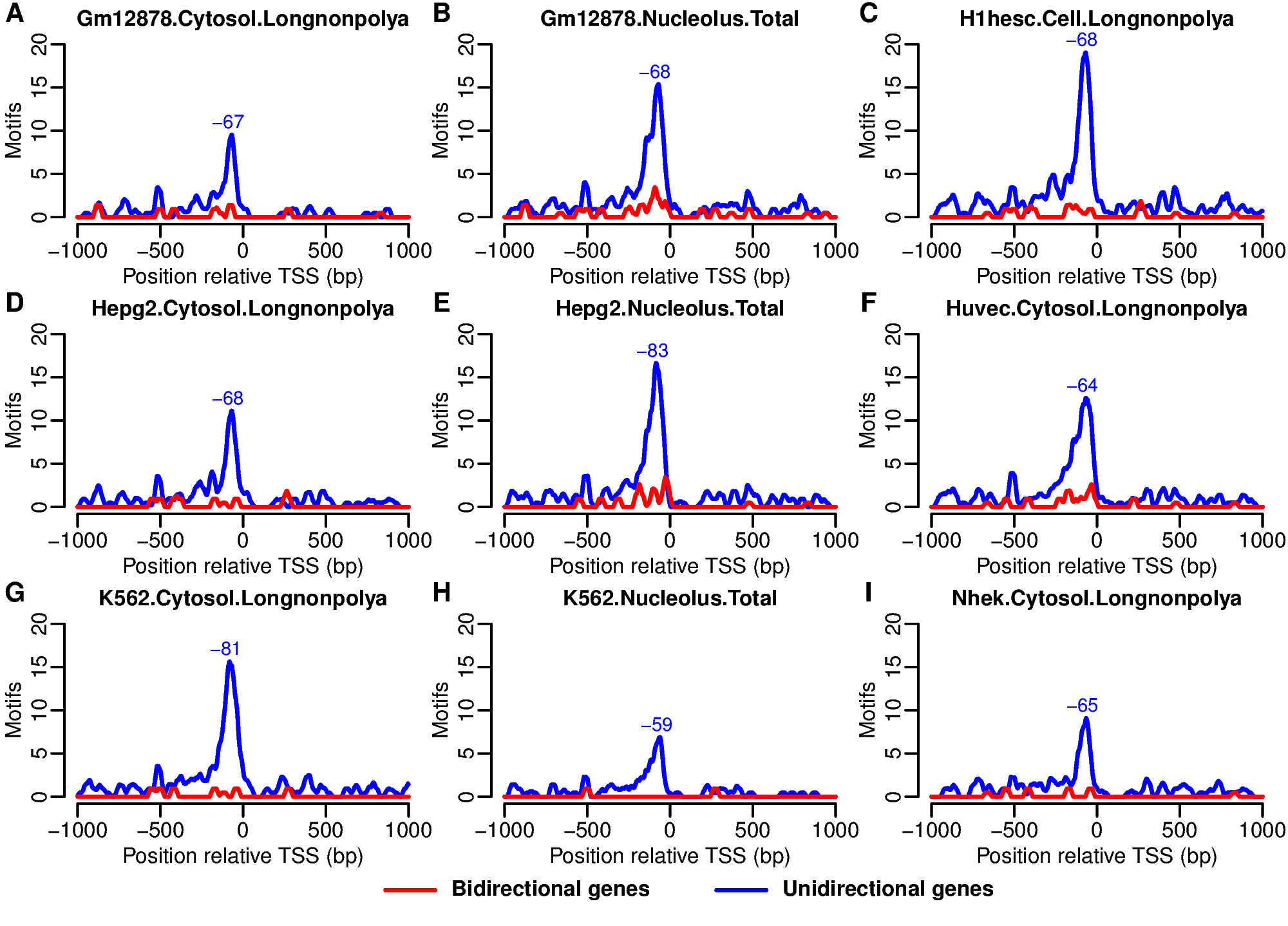


**Figure S10.** Position of the CTCF motif. The subfigure headers indicate cell line and subcellular origin of the CAGE data used for gene annotation. The per-bp motif coverage was computed in a region ±1 kb from the TSS for uni- and bidirectional genes separately. The signal shown was averaged over a ±20bp window and the position with the highest motif enrichment marked.

**Table S2. Number of genes by expression bin.**

|  | **Bidirectional (Ensembl+CAGE)** | | | | **Unidirectional (Ensembl+CAGE)** | | | |
| --- | --- | --- | --- | --- | --- | --- | --- | --- |
|  | **Lowest** | **Mid-low** | **Mid-high** | **Highest** | **Lowest** | **Mid-low** | **Mid-high** | **Highest** |
| GM12878, Cytosol, PolyA- | 92 | 82 | 69 | 75 | 672 | 669 | 685 | 686 |
| GM12878, Nucleolus, Total | 187 | 153 | 144 | 157 | 952 | 955 | 973 | 936 |
| H1hESC, Cell, PolyA- | 183 | 191 | 175 | 179 | 1195 | 1128 | 1154 | 1167 |
| HepG2, Cytosol, PolyA- | 94 | 105 | 91 | 88 | 838 | 830 | 833 | 858 |
| HepG2, Nucleolus, Total | 171 | 126 | 126 | 138 | 961 | 946 | 960 | 963 |
| HUVEC, Cytosol, PolyA- | 160 | 136 | 151 | 156 | 994 | 968 | 954 | 978 |
| K562, Cytosol, PolyA- | 84 | 103 | 94 | 82 | 890 | 894 | 861 | 902 |
| K562, Nucleolus, Total | 97 | 72 | 93 | 73 | 581 | 506 | 500 | 498 |
| NHEK, Cytosol, PolyA- | 66 | 88 | 75 | 63 | 699 | 682 | 667 | 691 |

The genes were divided into four expression bins based on CAGE. The number of bi- and unidirectional genes, respectively, that falls into each of the bins is shown for all cell lines.
