# Supplementary figures and images for: Different distribution of histone modifications in genes with unidirectional and bidirectional transcription and a role of CTCF and cohesin in directing transcription
Source: BMC Genomics. 2015 Apr 15;16(1):300. doi: 10.1186/s12864-015-1485-5 (PMC4446127; doi:10.1186/s12864-015-1485-5)

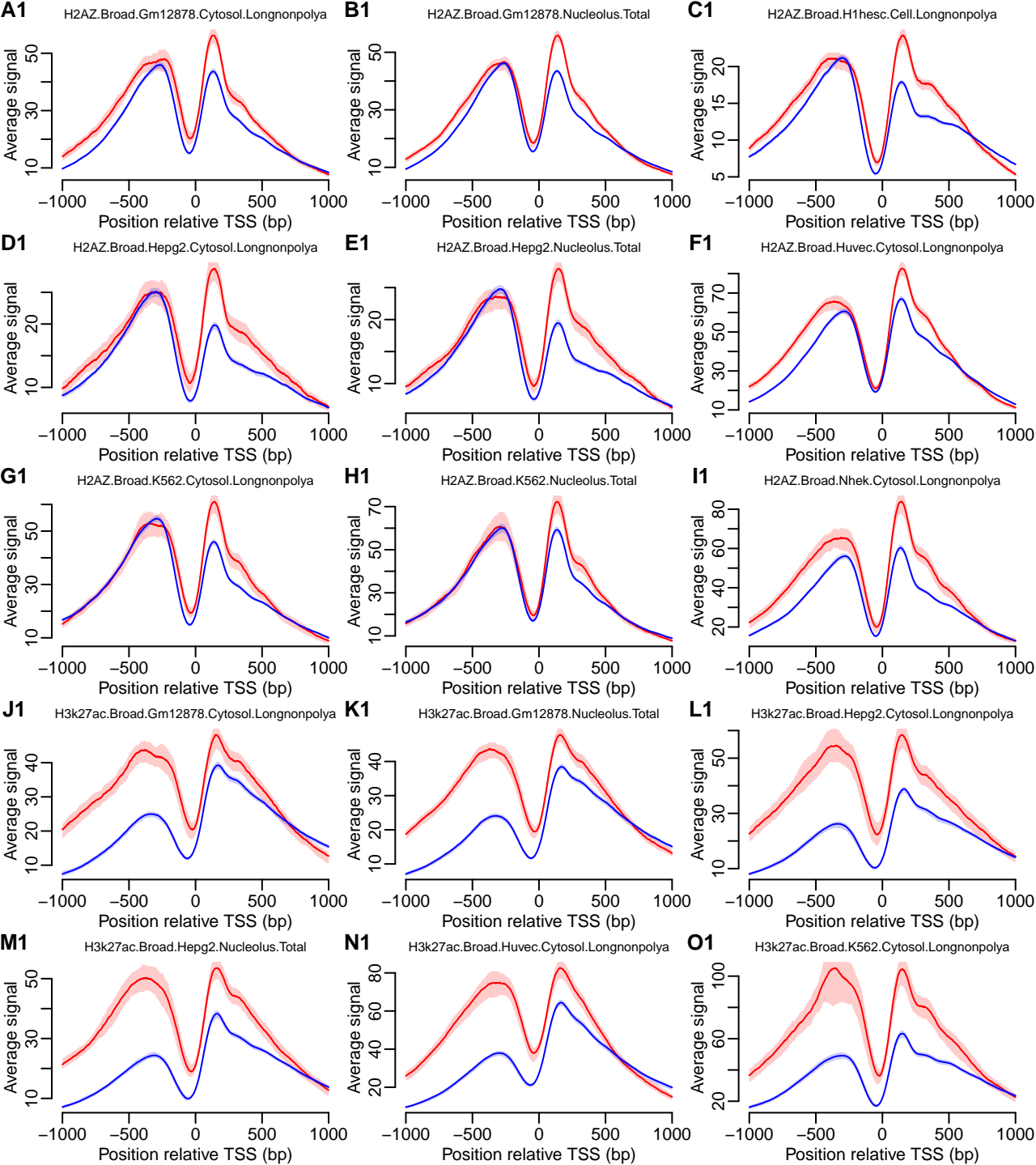

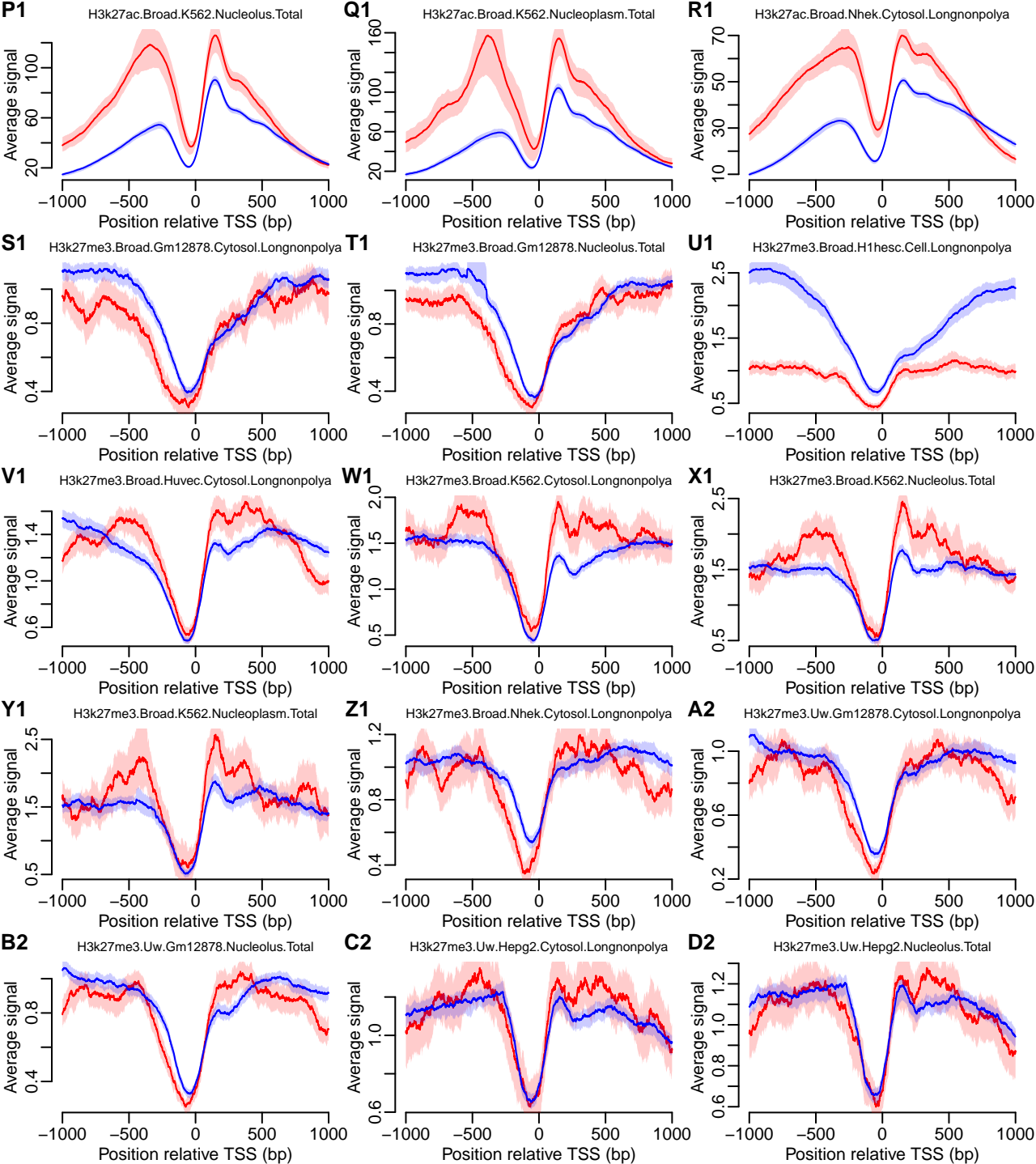

— Bidirectional genes

— Unidirectional genes

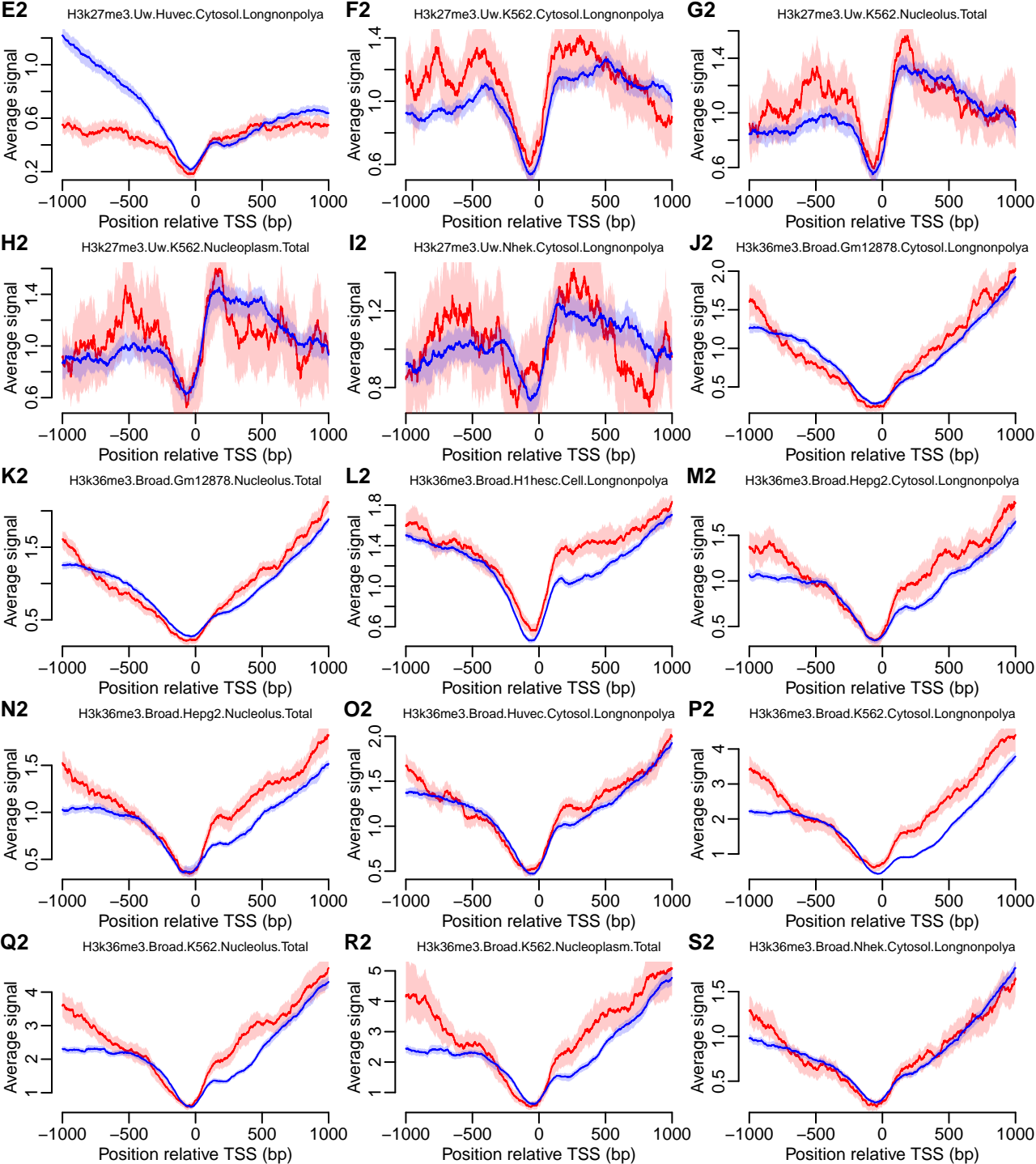

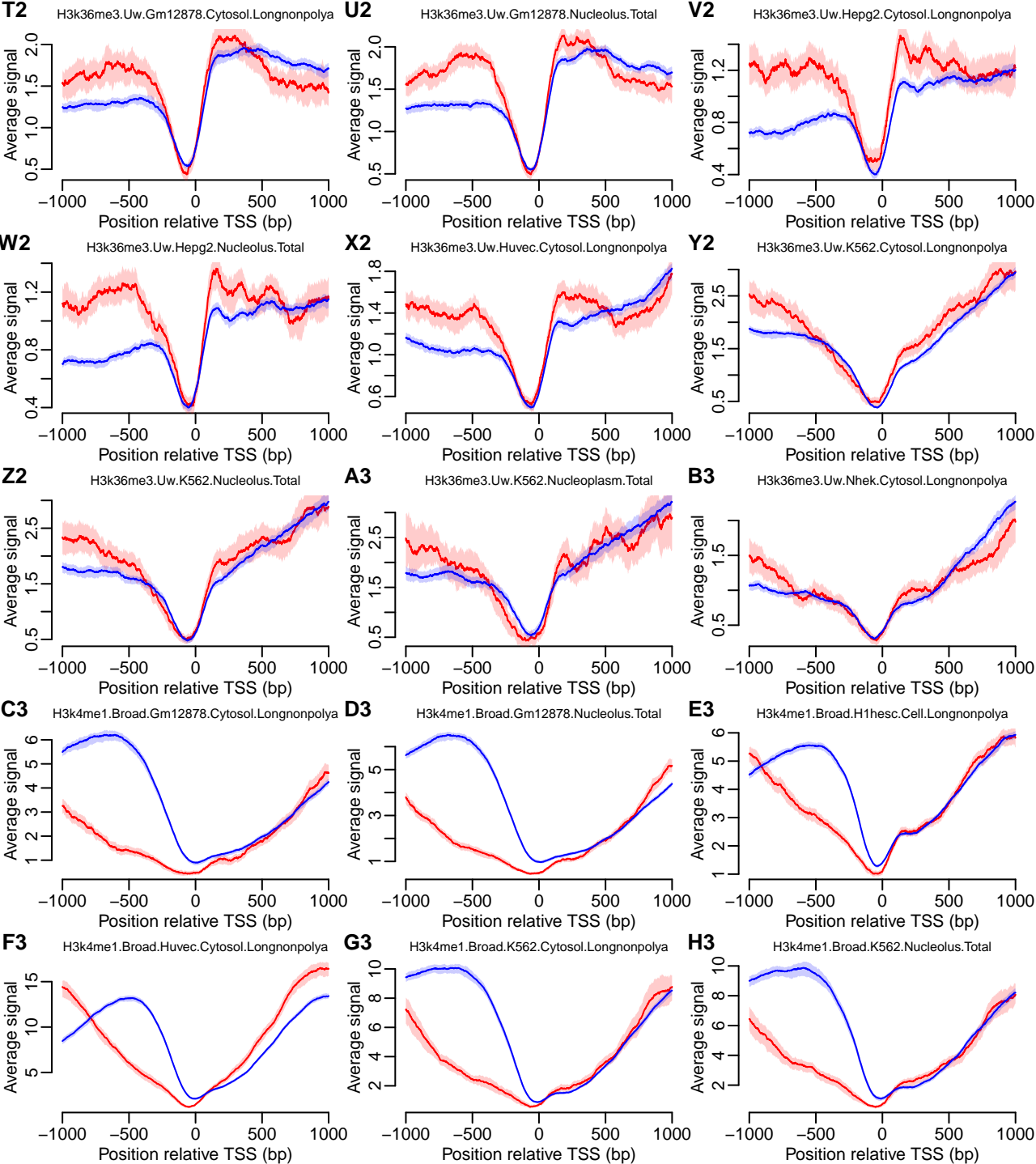

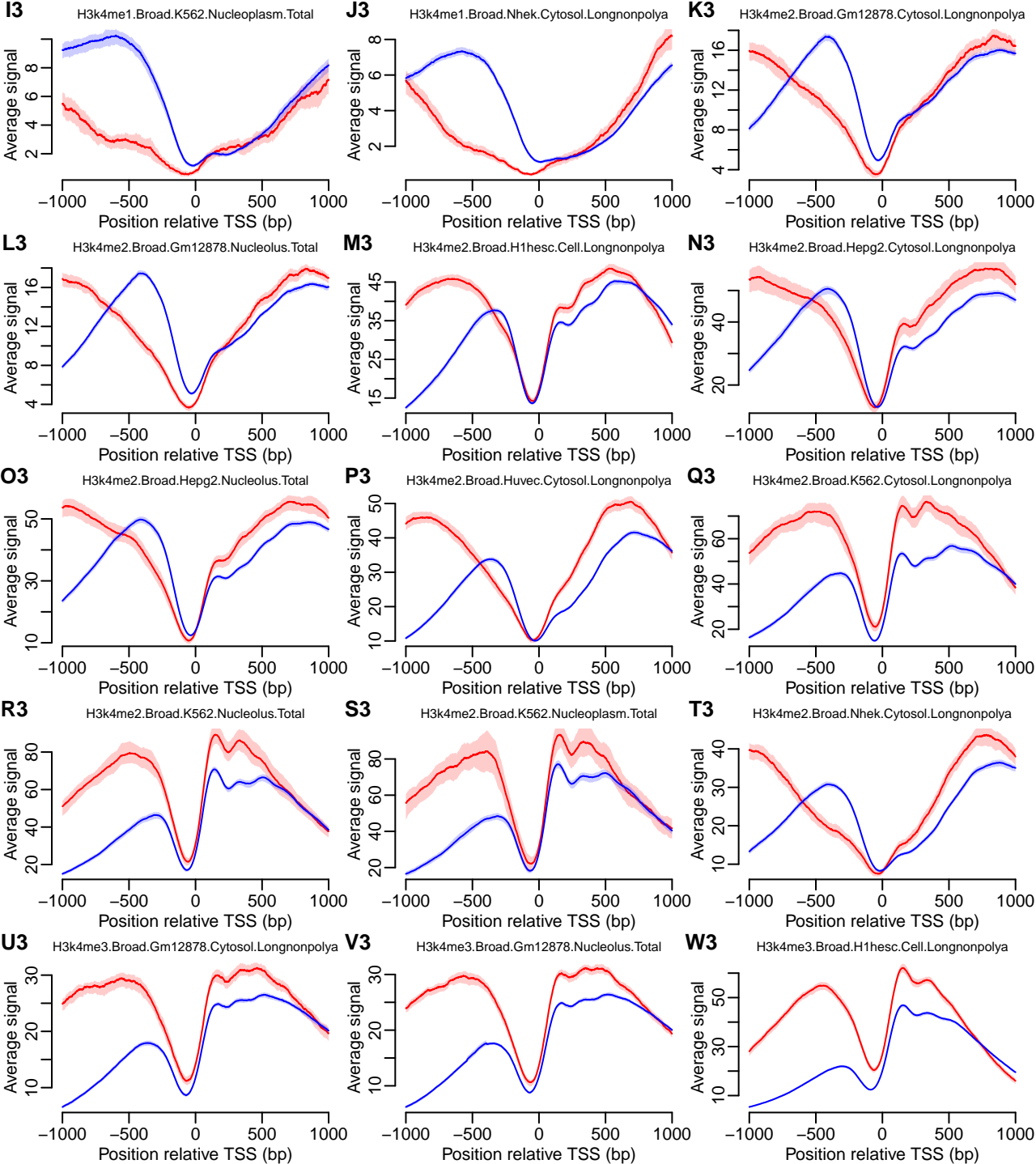

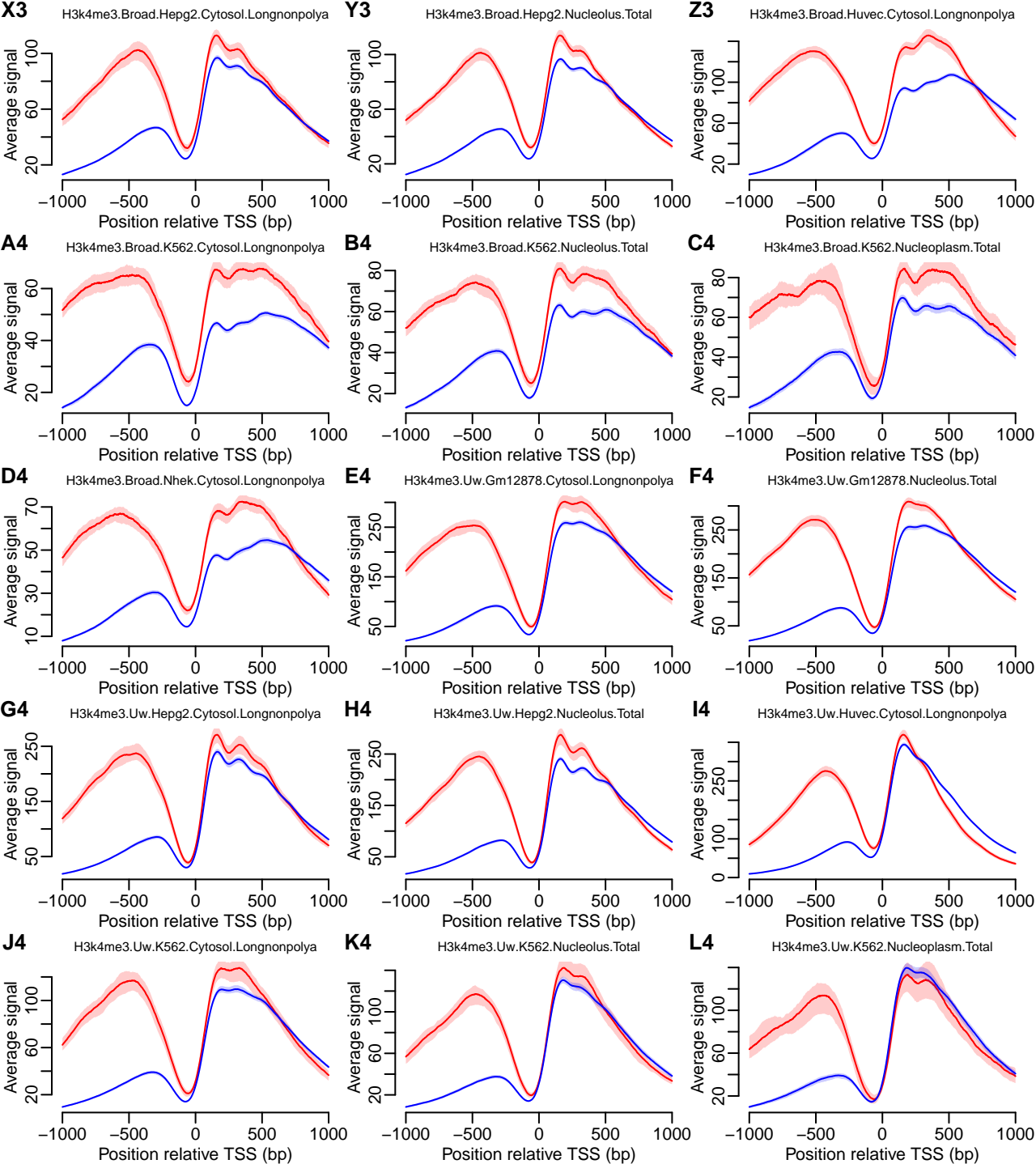

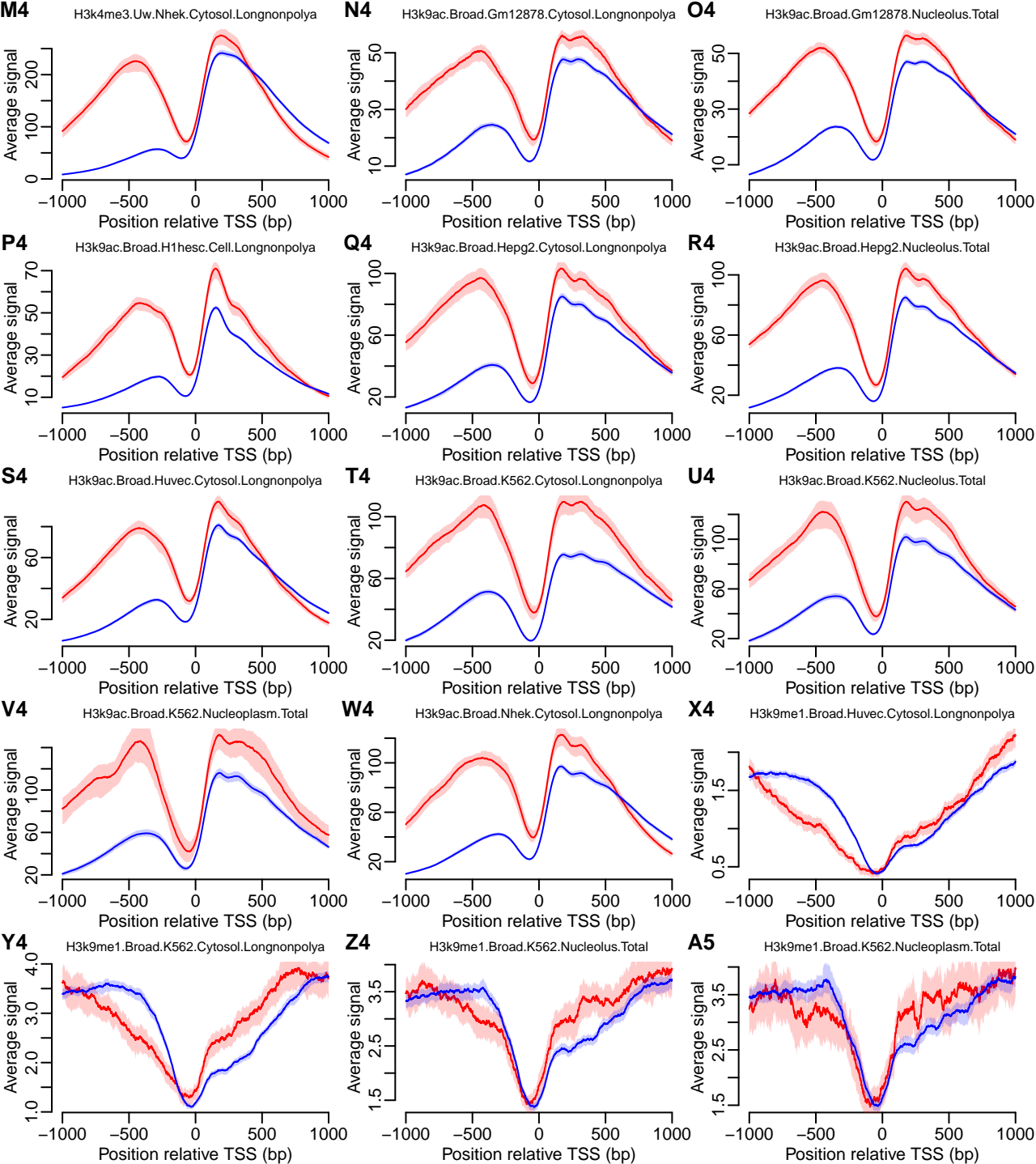

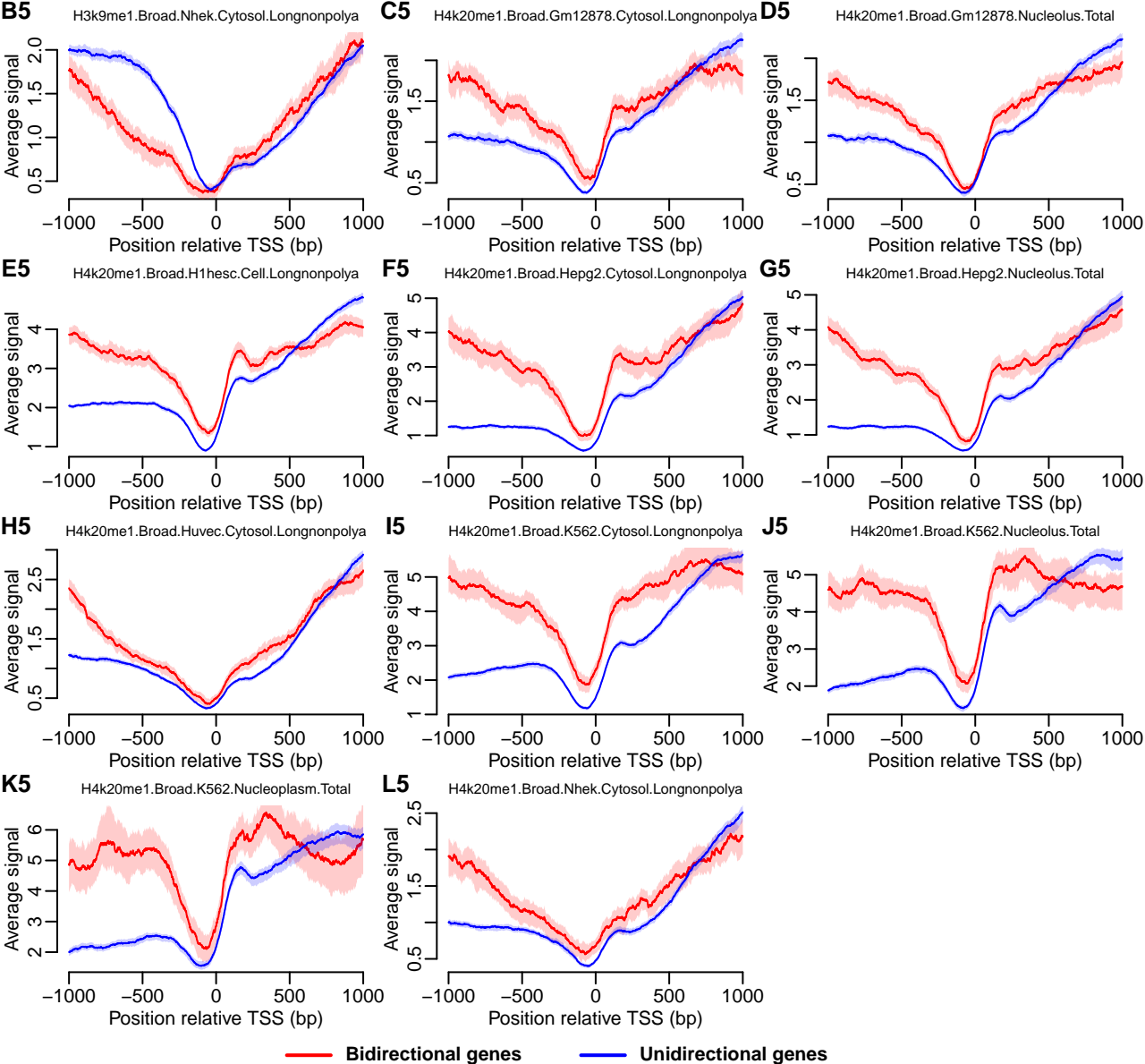

Supplement: Additional file 3: Figure S6. — Differences in HM signals between bi- and unidirectional genes annotated using both Ensembl and CAGE shown for all cell lines and 13 HM and histone variant datasets. The average signal (with 95% CI) is shown in a region ±1 kb from the TSS. [file 12864_2015_1485_MOESM3_ESM.pdf]
