# Supplementary material for: Different distribution of histone modifications in genes with unidirectional and bidirectional transcription and a role of CTCF and cohesin in directing transcription
Source: BMC Genomics. 2015 Apr 15;16(1):300. doi: 10.1186/s12864-015-1485-5 (PMC4446127; doi:10.1186/s12864-015-1485-5)
Supplement: Additional file 4: Figure S7. — Differences in TF signals between bi- and unidirectional genes annotated using both Ensembl and CAGE shown for all cell lines and 83 TF datasets. The average signal (with 95% CI) is shown in a region ±1 kb from the TSS. [file 12864_2015_1485_MOESM4_ESM.pdf]

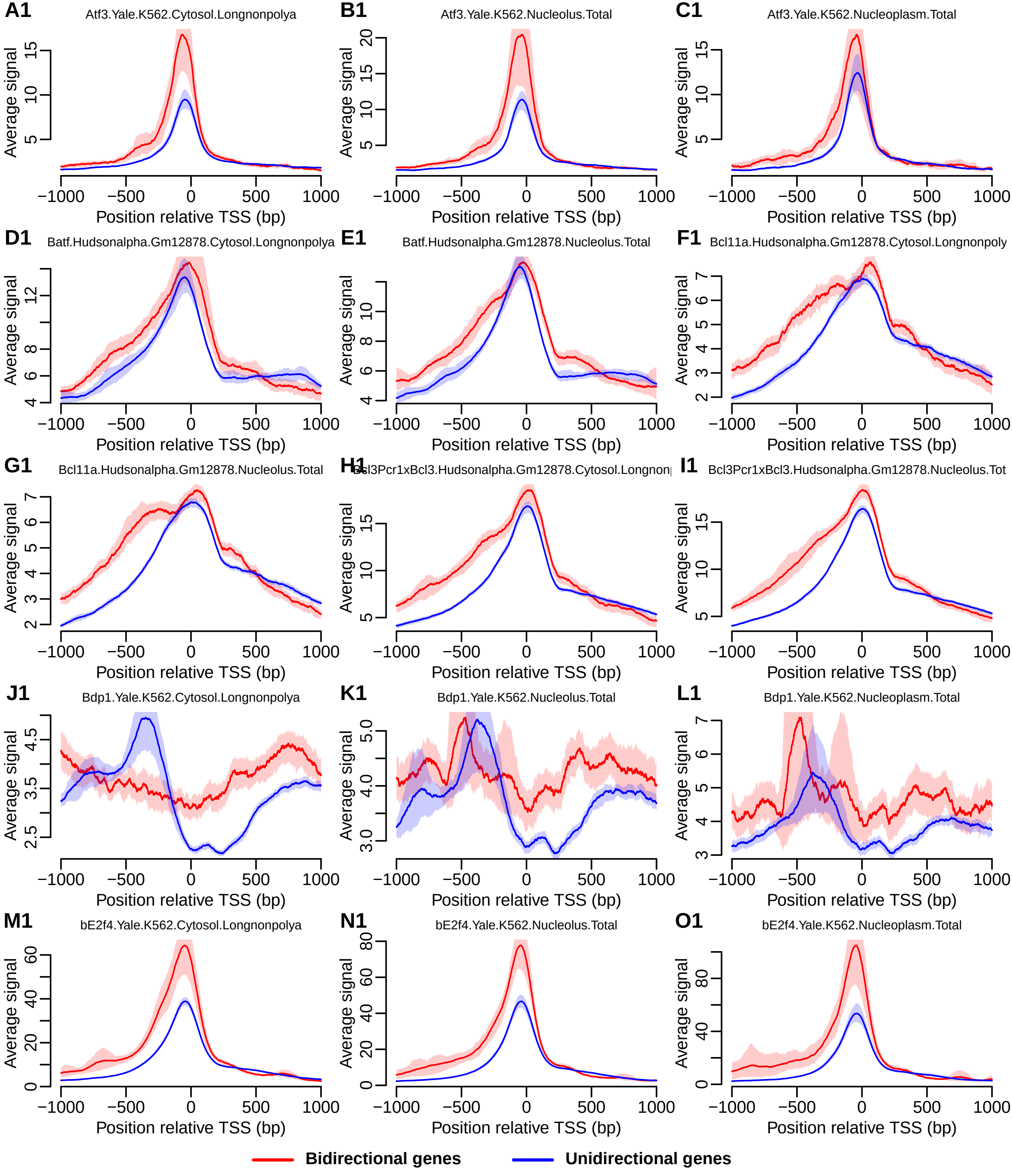

A1 Atf3.Yale.K562.Cytosol.Longnonpolya
B1 Atf3.Yale.K562.Nucleolus.Total
C1 Atf3.Yale.K562.Nucleoplasm.Total
D1 Batf.Hudsonalpha.Gm12878.Cytosol.Longnonpolya
E1 Batf.Hudsonalpha.Gm12878.Nucleolus.Total
F1 Bcl11a.Hudsonalpha.Gm12878.Cytosol.Longnonpoly
G1 Bcl11a.Hudsonalpha.Gm12878.Nucleolus.Total
H1 Bcl3Pcr1xBcl3.Hudsonalpha.Gm12878.Cytosol.Longnon
I1 Bcl3Pcr1xBcl3.Hudsonalpha.Gm12878.Nucleolus.Tot
J1 Bdp1.Yale.K562.Cytosol.Longnonpolya
K1 Bdp1.Yale.K562.Nucleolus.Total
L1 Bdp1.Yale.K562.Nucleoplasm.Total
M1 bE2f4.Yale.K562.Cytosol.Longnonpolya
N1 bE2f4.Yale.K562.Nucleolus.Total
O1 bE2f4.Yale.K562.Nucleoplasm.Total
Average signal
Position relative TSS (bp)
−1000 −500 0 500 1000
Bidirectional genes
Unidirectional genes

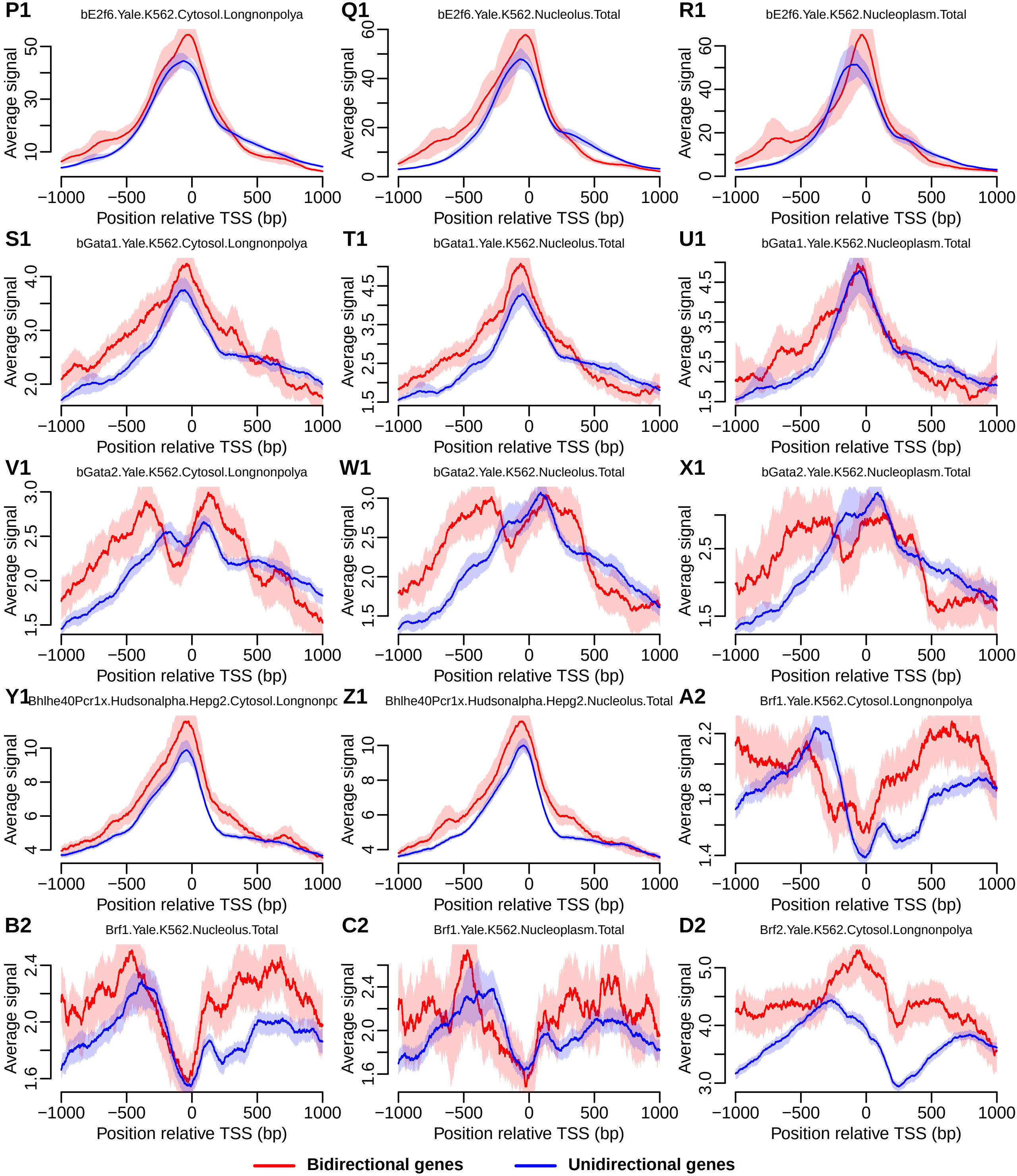

P1
bE2f6.Yale.K562.Cytosol.Longnonpolya
Q1
bE2f6.Yale.K562.Nucleolus.Total
R1
bE2f6.Yale.K562.Nucleoplasm.Total
S1
bGata1.Yale.K562.Cytosol.Longnonpolya
T1
bGata1.Yale.K562.Nucleolus.Total
U1
bGata1.Yale.K562.Nucleoplasm.Total
V1
bGata2.Yale.K562.Cytosol.Longnonpolya
W1
bGata2.Yale.K562.Nucleolus.Total
X1
bGata2.Yale.K562.Nucleoplasm.Total
Y1
Bhlhe40Pcr1x.Hudsonalpha.Hepg2.Cytosol.Longnonpc
Z1
Bhlhe40Pcr1x.Hudsonalpha.Hepg2.Nucleolus.Total
A2
Brf1.Yale.K562.Cytosol.Longnonpolya
B2
Brf1.Yale.K562.Nucleolus.Total
C2
Brf1.Yale.K562.Nucleoplasm.Total
D2
Brf2.Yale.K562.Cytosol.Longnonpolya
Average signal
Position relative TSS (bp)
−1000
−500
0
500
1000
Bidirectional genes
Unidirectional genes

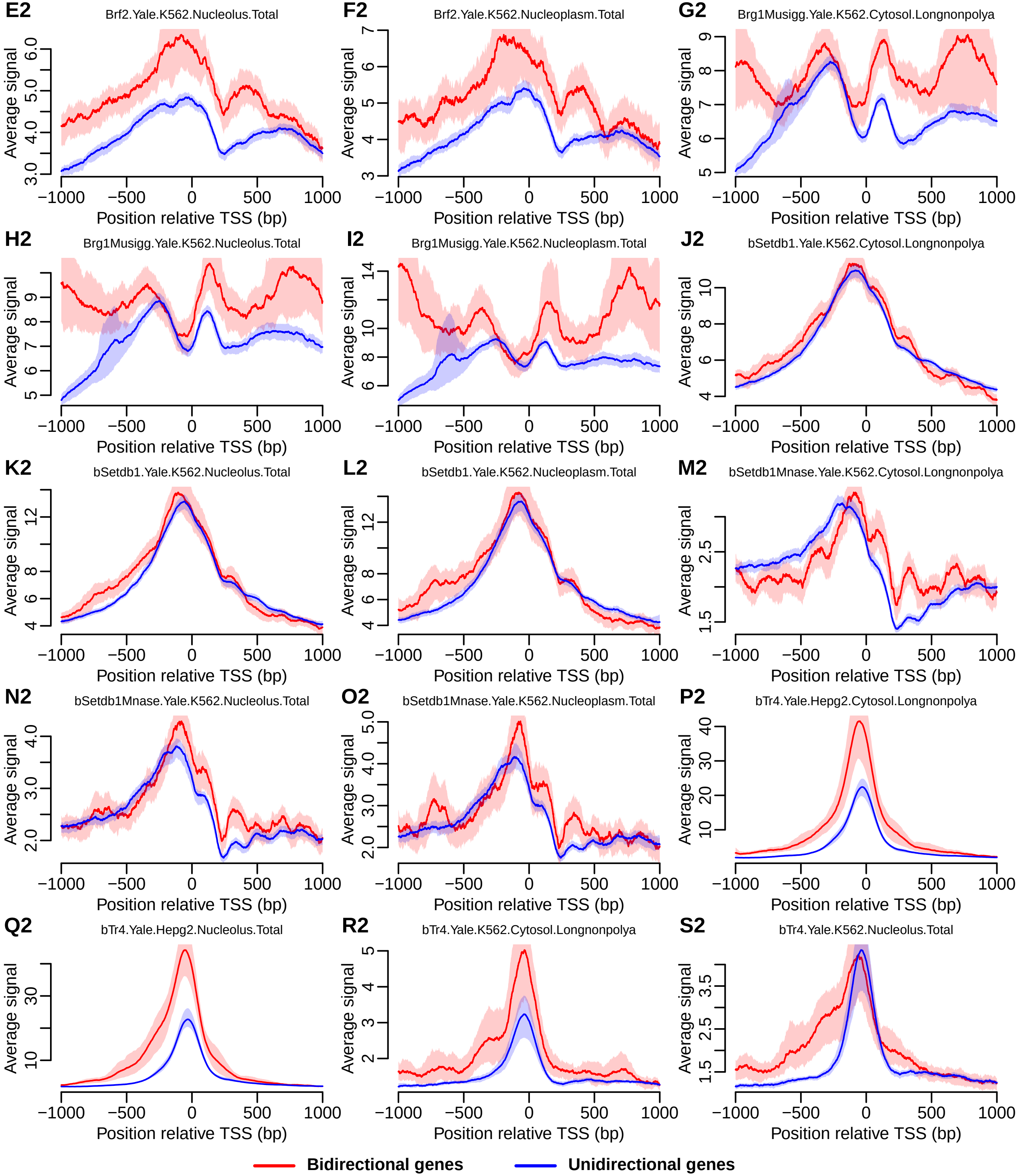
E2
Brf2.Yale.K562.Nucleolus.Total
F2
Brf2.Yale.K562.Nucleoplasm.Total
G2
Brg1Musigg.Yale.K562.Cytosol.Longnonpolya
H2
Brg1Musigg.Yale.K562.Nucleolus.Total
I2
Brg1Musigg.Yale.K562.Nucleoplasm.Total
J2
bSetdb1.Yale.K562.Cytosol.Longnonpolya
K2
bSetdb1.Yale.K562.Nucleolus.Total
L2
bSetdb1.Yale.K562.Nucleoplasm.Total
M2
bSetdb1Mnase.Yale.K562.Cytosol.Longnonpolya
N2
bSetdb1Mnase.Yale.K562.Nucleolus.Total
O2
bSetdb1Mnase.Yale.K562.Nucleoplasm.Total
P2
bTr4.Yale.Hepg2.Cytosol.Longnonpolya
Q2
bTr4.Yale.Hepg2.Nucleolus.Total
R2
bTr4.Yale.K562.Cytosol.Longnonpolya
S2
bTr4.Yale.K562.Nucleolus.Total
Average signal
Position relative TSS (bp)
−1000
−500
0
500
1000
Bidirectional genes
Unidirectional genes

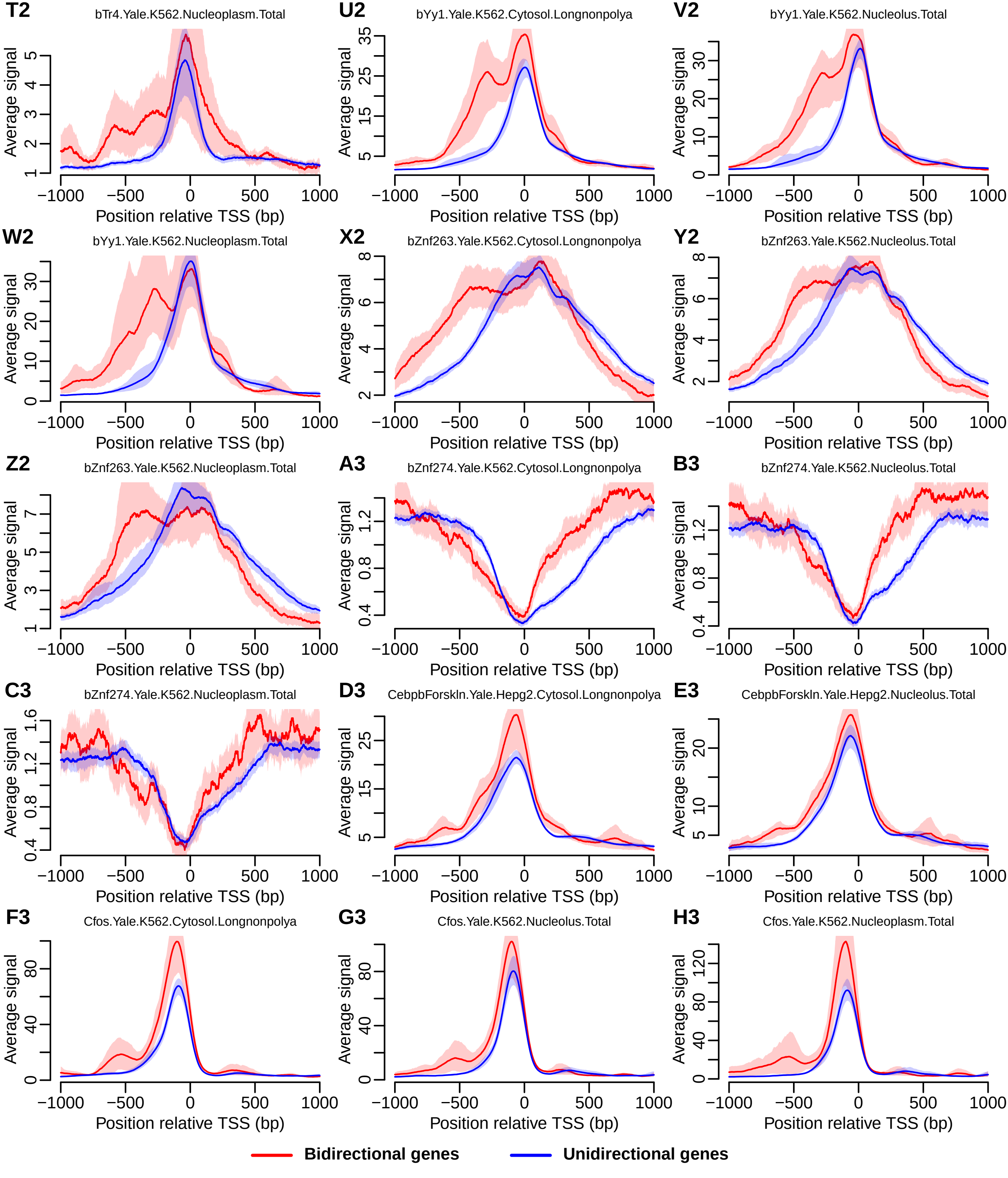

T2
bTr4.Yale.K562.Nucleoplasm.Total
U2
bYy1.Yale.K562.Cytosol.Longnonpolya
V2
bYy1.Yale.K562.Nucleolus.Total
W2
bYy1.Yale.K562.Nucleoplasm.Total
X2
bZnf263.Yale.K562.Cytosol.Longnonpolya
Y2
bZnf263.Yale.K562.Nucleolus.Total
Z2
bZnf263.Yale.K562.Nucleoplasm.Total
A3
bZnf274.Yale.K562.Cytosol.Longnonpolya
B3
bZnf274.Yale.K562.Nucleolus.Total
C3
bZnf274.Yale.K562.Nucleoplasm.Total
D3
CebpbForskln.Yale.Hepg2.Cytosol.Longnonpolya
E3
CebpbForskln.Yale.Hepg2.Nucleolus.Total
F3
Cfos.Yale.K562.Cytosol.Longnonpolya
G3
Cfos.Yale.K562.Nucleolus.Total
H3
Cfos.Yale.K562.Nucleoplasm.Total
Average signal
Position relative TSS (bp)
−1000 −500 0 500 1000
Bidirectional genes
Unidirectional genes

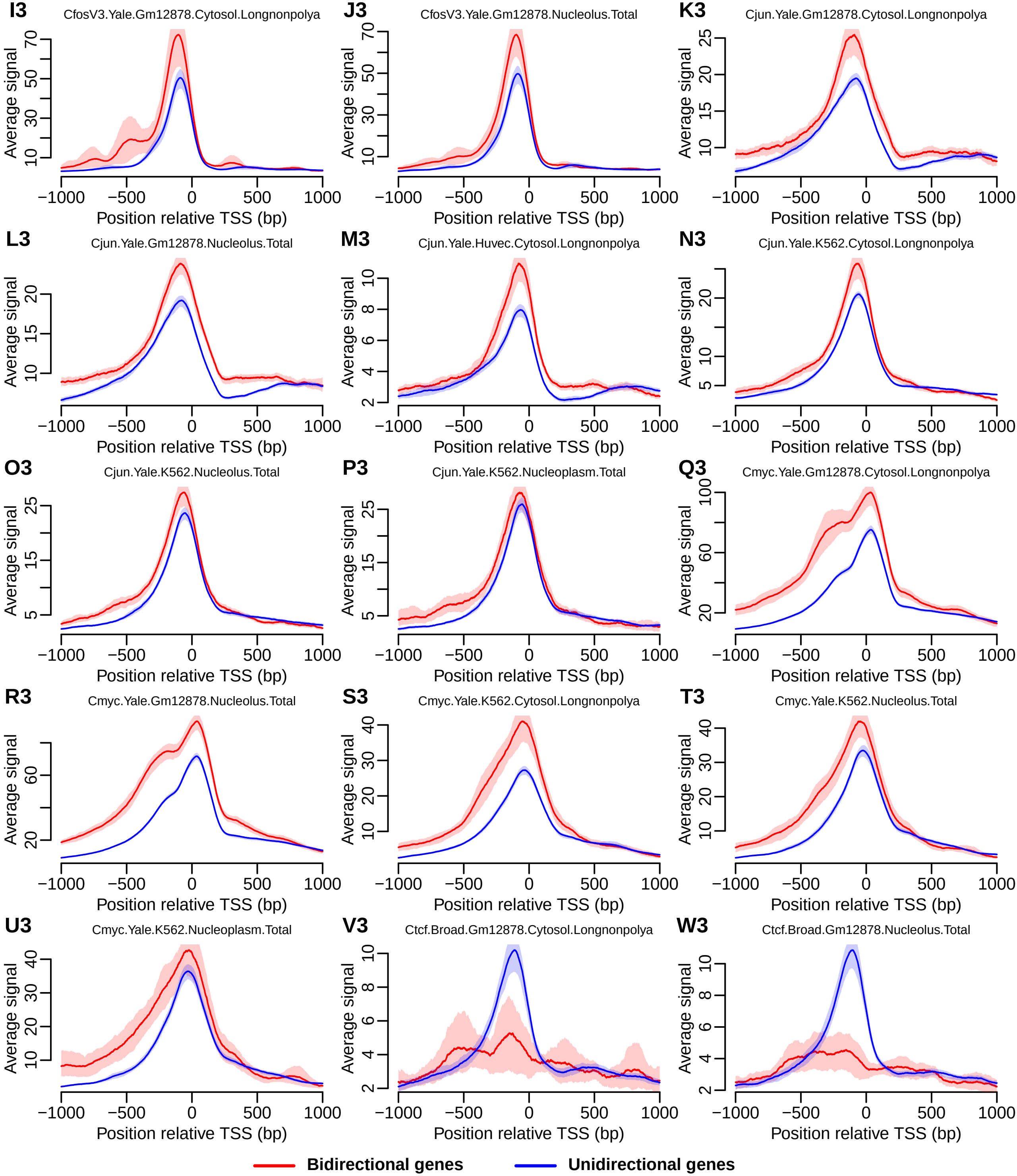

I3
CfosV3.Yale.Gm12878.Cytosol.Longnonpolya
J3
CfosV3.Yale.Gm12878.Nucleolus.Total
K3
Cjun.Yale.Gm12878.Cytosol.Longnonpolya
L3
Cjun.Yale.Gm12878.Nucleolus.Total
M3
Cjun.Yale.Huvec.Cytosol.Longnonpolya
N3
Cjun.Yale.K562.Cytosol.Longnonpolya
O3
Cjun.Yale.K562.Nucleolus.Total
P3
Cjun.Yale.K562.Nucleoplasm.Total
Q3
Cmyc.Yale.Gm12878.Cytosol.Longnonpolya
R3
Cmyc.Yale.Gm12878.Nucleolus.Total
S3
Cmyc.Yale.K562.Cytosol.Longnonpolya
T3
Cmyc.Yale.K562.Nucleolus.Total
U3
Cmyc.Yale.K562.Nucleoplasm.Total
V3
Ctcf.Broad.Gm12878.Cytosol.Longnonpolya
W3
Ctcf.Broad.Gm12878.Nucleolus.Total
Average signal
Position relative TSS (bp)
−1000
−500
0
500
1000
Bidirectional genes
Unidirectional genes

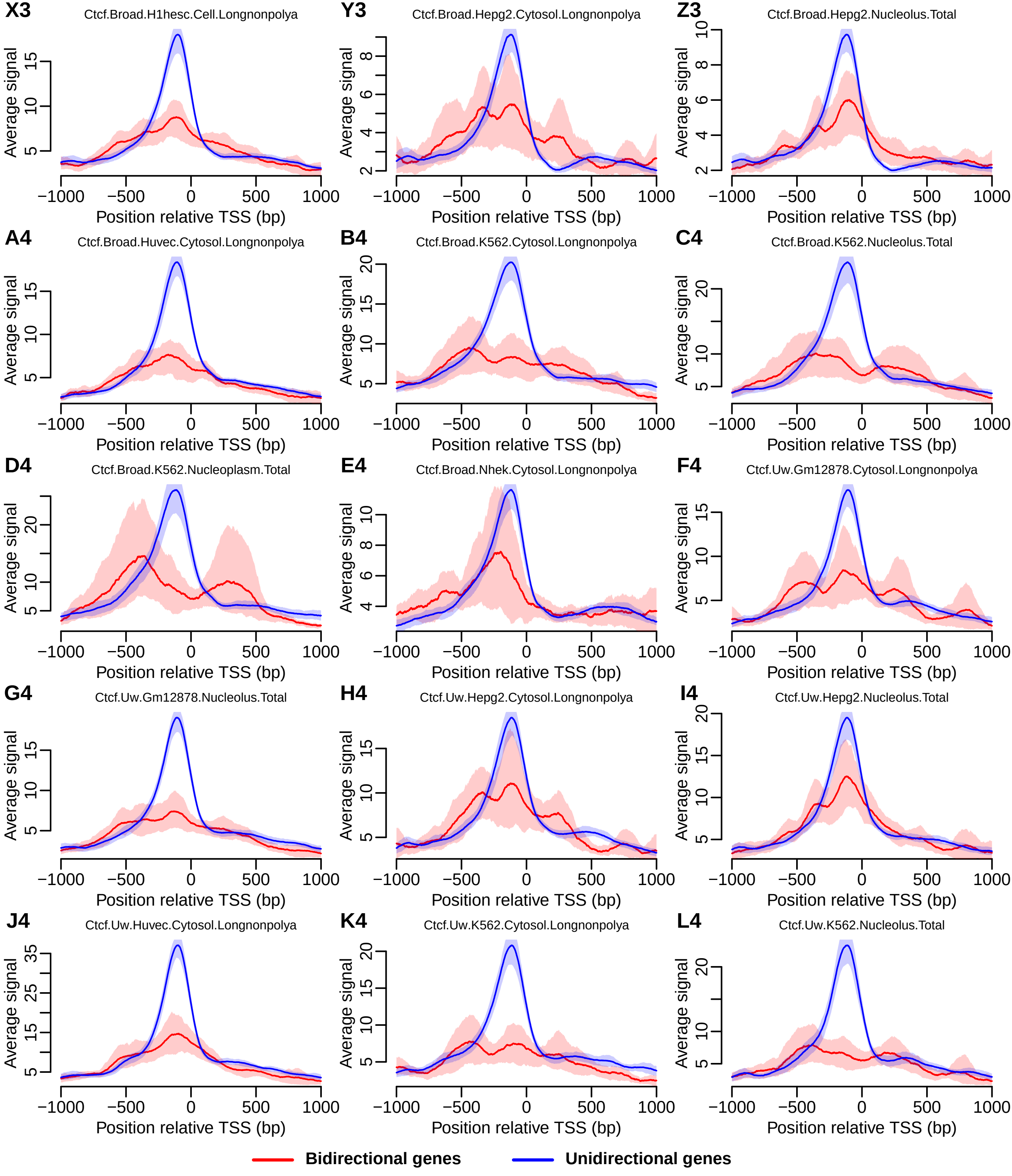
X3
Ctcf.Broad.H1hesc.Cell.Longnonpolya
Y3
Ctcf.Broad.Hepg2.Cytosol.Longnonpolya
Z3
Ctcf.Broad.Hepg2.Nucleolus.Total
A4
Ctcf.Broad.Huvec.Cytosol.Longnonpolya
B4
Ctcf.Broad.K562.Cytosol.Longnonpolya
C4
Ctcf.Broad.K562.Nucleolus.Total
D4
Ctcf.Broad.K562.Nucleoplasm.Total
E4
Ctcf.Broad.Nhek.Cytosol.Longnonpolya
F4
Ctcf.Uw.Gm12878.Cytosol.Longnonpolya
G4
Ctcf.Uw.Gm12878.Nucleolus.Total
H4
Ctcf.Uw.Hepg2.Cytosol.Longnonpolya
I4
Ctcf.Uw.Hepg2.Nucleolus.Total
J4
Ctcf.Uw.Huvec.Cytosol.Longnonpolya
K4
Ctcf.Uw.K562.Cytosol.Longnonpolya
L4
Ctcf.Uw.K562.Nucleolus.Total
Average signal
Position relative TSS (bp)
−1000 −500 0 500 1000
Bidirectional genes
Unidirectional genes

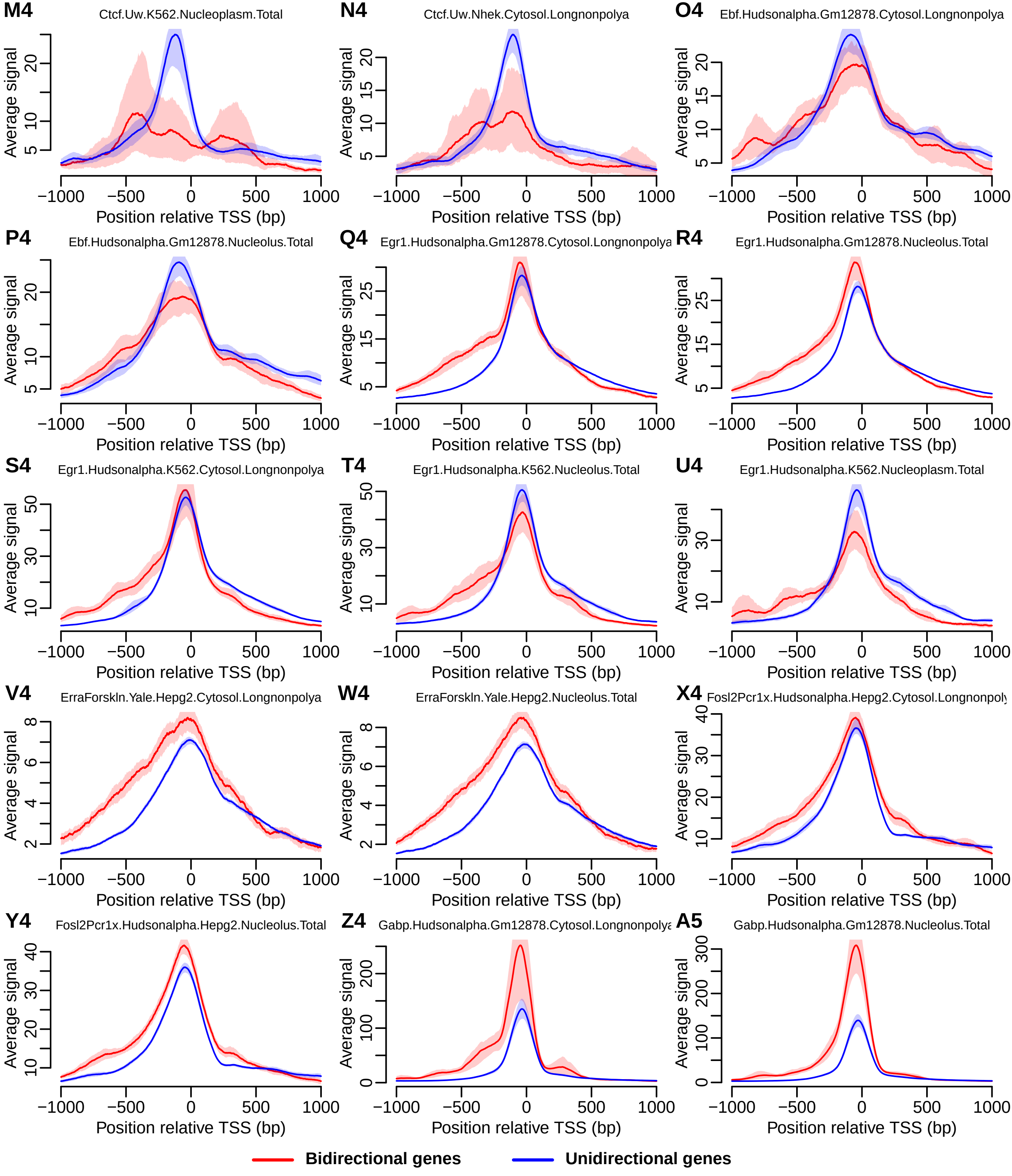
M4 Ctcf.Uw.K562.Nucleoplasm.Total
N4 Ctcf.Uw.Nhek.Cytosol.Longnonpolya
O4 Ebf.Hudsonalpha.Gm12878.Cytosol.Longnonpolya
P4 Ebf.Hudsonalpha.Gm12878.Nucleolus.Total
Q4 Egr1.Hudsonalpha.Gm12878.Cytosol.Longnonpolya
R4 Egr1.Hudsonalpha.Gm12878.Nucleolus.Total
S4 Egr1.Hudsonalpha.K562.Cytosol.Longnonpolya
T4 Egr1.Hudsonalpha.K562.Nucleolus.Total
U4 Egr1.Hudsonalpha.K562.Nucleoplasm.Total
V4 ErraForskln.Yale.Hepg2.Cytosol.Longnonpolya
W4 ErraForskln.Yale.Hepg2.Nucleolus.Total
X4 Fosl2Pcr1x.Hudsonalpha.Hepg2.Cytosol.Longnonpoly
Y4 Fosl2Pcr1x.Hudsonalpha.Hepg2.Nucleolus.Total
Z4 Gabp.Hudsonalpha.Gm12878.Cytosol.Longnonpolya
A5 Gabp.Hudsonalpha.Gm12878.Nucleolus.Total
Average signal
Position relative TSS (bp)
Bidirectional genes
Unidirectional genes

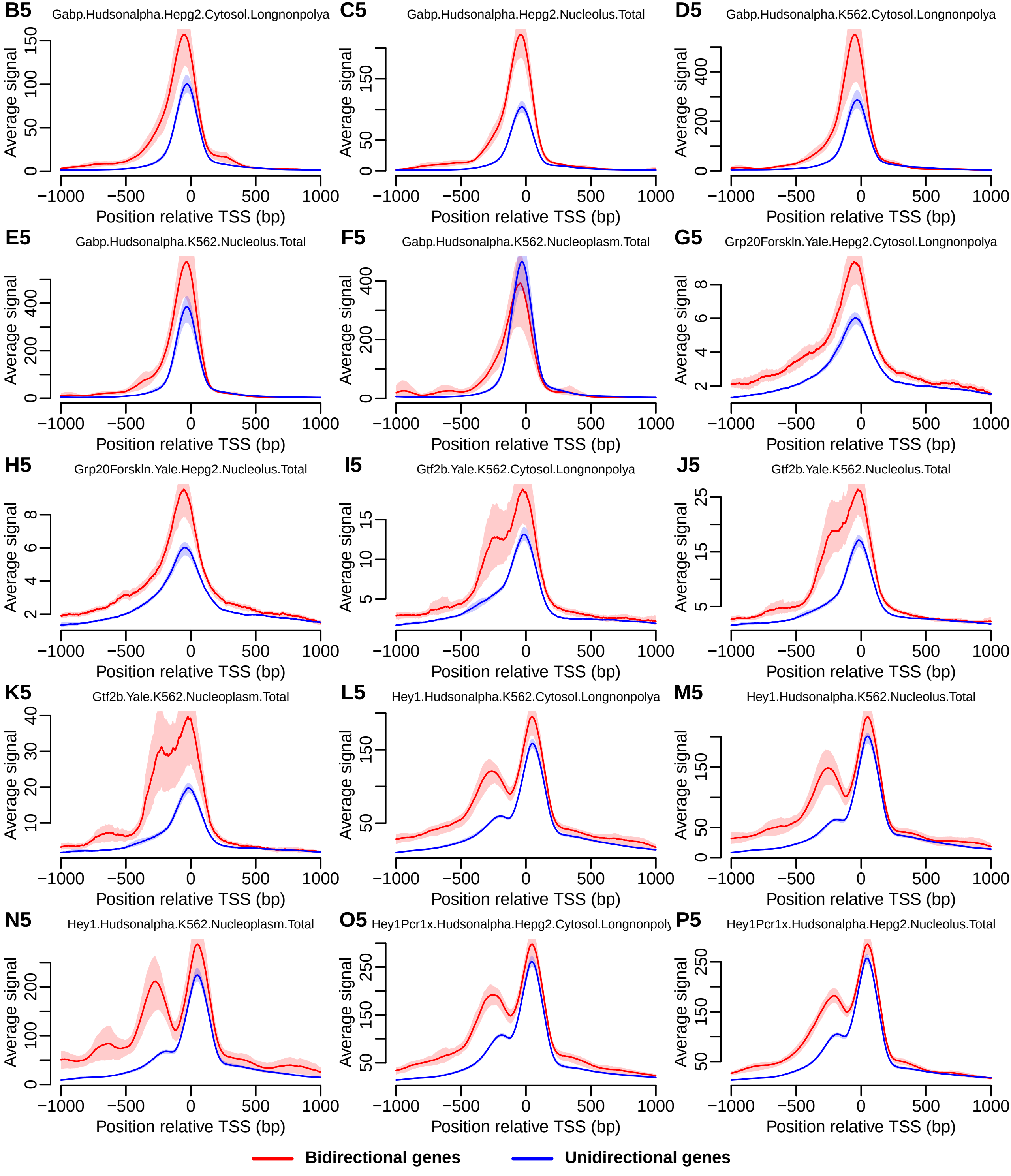

B5
Gabp.Hudsonalpha.Hepg2.Cytosol.Longnonpolya
C5
Gabp.Hudsonalpha.Hepg2.Nucleolus.Total
D5
Gabp.Hudsonalpha.K562.Cytosol.Longnonpolya
E5
Gabp.Hudsonalpha.K562.Nucleolus.Total
F5
Gabp.Hudsonalpha.K562.Nucleoplasm.Total
G5
Grp20Forskln.Yale.Hepg2.Cytosol.Longnonpolya
H5
Grp20Forskln.Yale.Hepg2.Nucleolus.Total
I5
Gtf2b.Yale.K562.Cytosol.Longnonpolya
J5
Gtf2b.Yale.K562.Nucleolus.Total
K5
Gtf2b.Yale.K562.Nucleoplasm.Total
L5
Hey1.Hudsonalpha.K562.Cytosol.Longnonpolya
M5
Hey1.Hudsonalpha.K562.Nucleolus.Total
N5
Hey1.Hudsonalpha.K562.Nucleoplasm.Total
O5
Hey1Pcr1x.Hudsonalpha.Hepg2.Cytosol.Longnonpoly
P5
Hey1Pcr1x.Hudsonalpha.Hepg2.Nucleolus.Total
Average signal
Position relative TSS (bp)
−1000 −500 0 500 1000
Bidirectional genes
Unidirectional genes

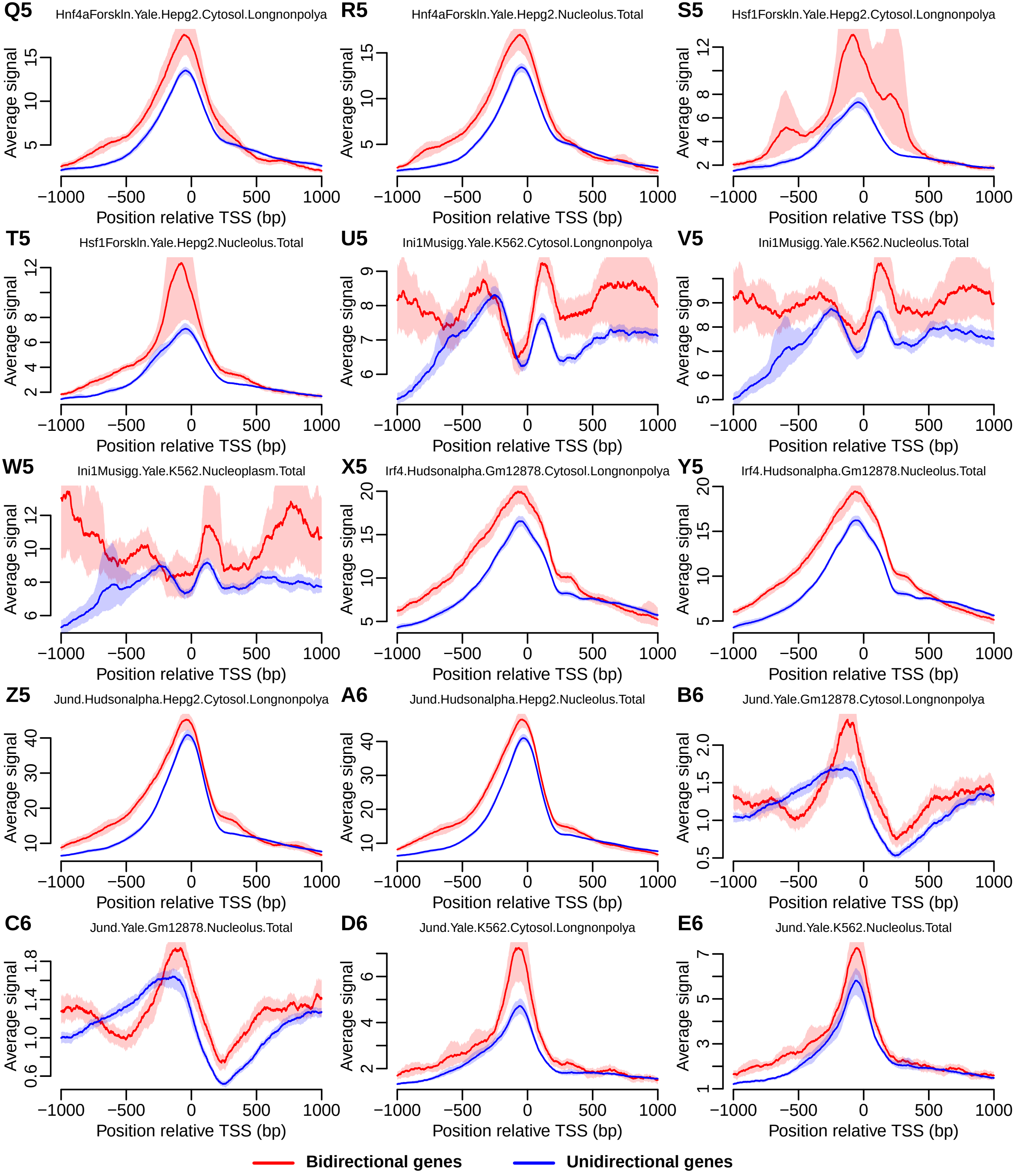
Q5
Hnf4aForskln.Yale.Hepg2.Cytosol.Longnonpolya
R5
Hnf4aForskln.Yale.Hepg2.Nucleolus.Total
S5
Hsf1Forskln.Yale.Hepg2.Cytosol.Longnonpolya
T5
Hsf1Forskln.Yale.Hepg2.Nucleolus.Total
U5
Ini1Musigg.Yale.K562.Cytosol.Longnonpolya
V5
Ini1Musigg.Yale.K562.Nucleolus.Total
W5
Ini1Musigg.Yale.K562.Nucleoplasm.Total
X5
Irf4.Hudsonalpha.Gm12878.Cytosol.Longnonpolya
Y5
Irf4.Hudsonalpha.Gm12878.Nucleolus.Total
Z5
Jund.Hudsonalpha.Hepg2.Cytosol.Longnonpolya
A6
Jund.Hudsonalpha.Hepg2.Nucleolus.Total
B6
Jund.Yale.Gm12878.Cytosol.Longnonpolya
C6
Jund.Yale.Gm12878.Nucleolus.Total
D6
Jund.Yale.K562.Cytosol.Longnonpolya
E6
Jund.Yale.K562.Nucleolus.Total
Average signal
Position relative TSS (bp)
−1000 −500 0 500 1000
Bidirectional genes
Unidirectional genes

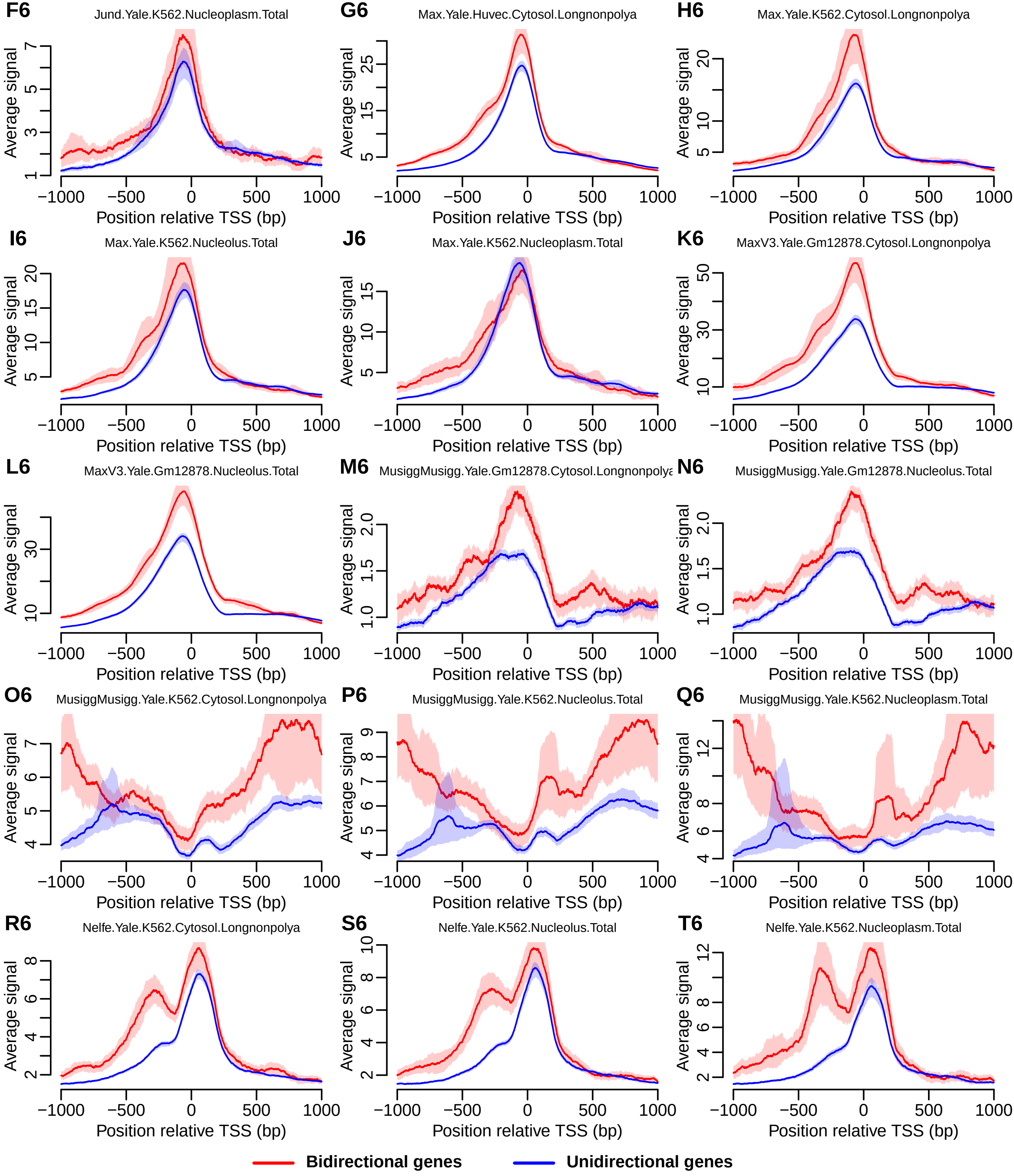
F6
Jund.Yale.K562.Nucleoplasm.Total
G6
Max.Yale.Huvec.Cytosol.Longnonpolya
H6
Max.Yale.K562.Cytosol.Longnonpolya
I6
Max.Yale.K562.Nucleolus.Total
J6
Max.Yale.K562.Nucleoplasm.Total
K6
MaxV3.Yale.Gm12878.Cytosol.Longnonpolya
L6
MaxV3.Yale.Gm12878.Nucleolus.Total
M6
MusiggMusigg.Yale.Gm12878.Cytosol.Longnonpolya
N6
MusiggMusigg.Yale.Gm12878.Nucleolus.Total
O6
MusiggMusigg.Yale.K562.Cytosol.Longnonpolya
P6
MusiggMusigg.Yale.K562.Nucleolus.Total
Q6
MusiggMusigg.Yale.K562.Nucleoplasm.Total
R6
Nelfe.Yale.K562.Cytosol.Longnonpolya
S6
Nelfe.Yale.K562.Nucleolus.Total
T6
Nelfe.Yale.K562.Nucleoplasm.Total
Average signal
Position relative TSS (bp)
−1000 −500 0 500 1000
Bidirectional genes
Unidirectional genes

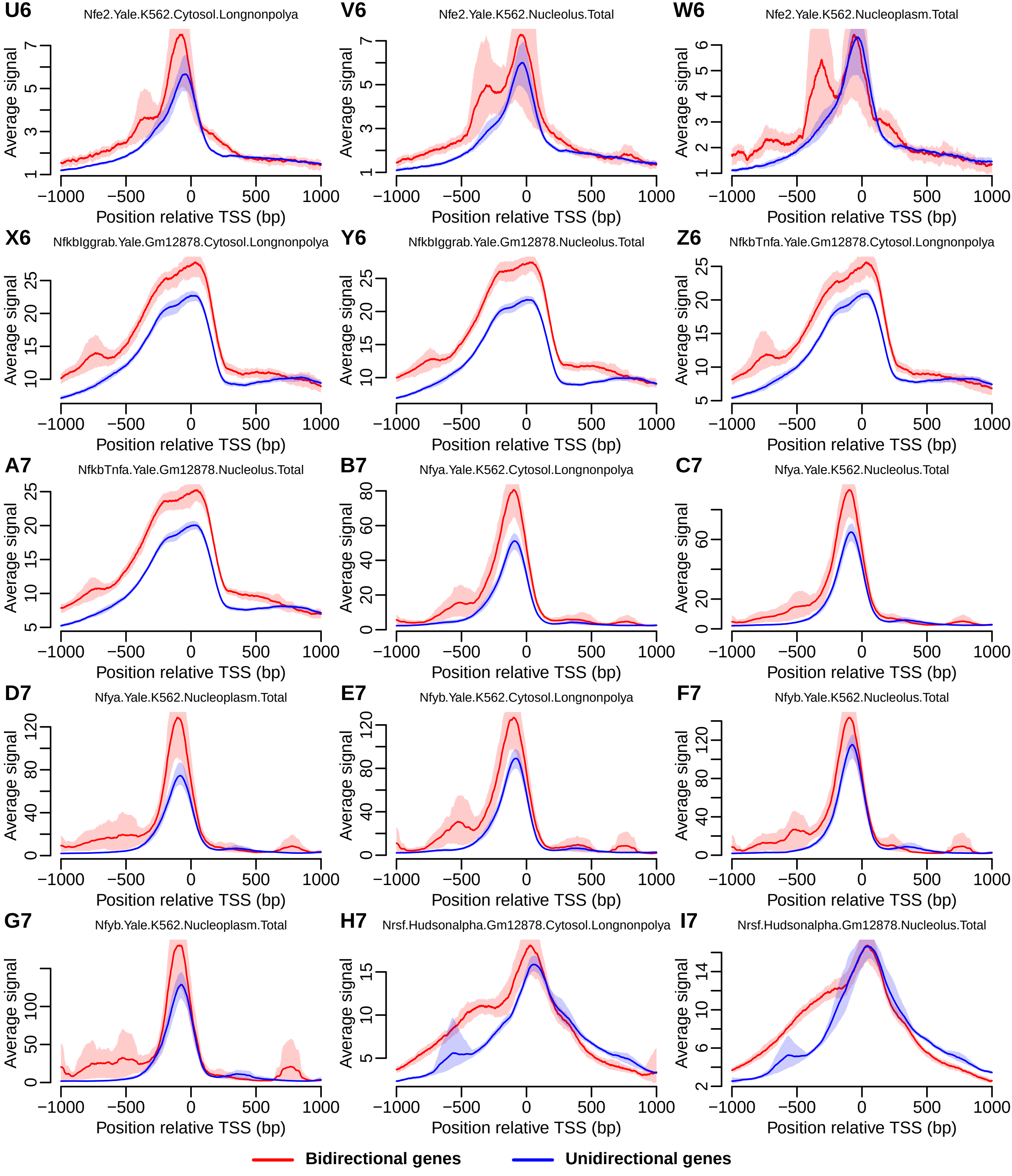
U6
Nfe2.Yale.K562.Cytosol.Longnonpolya
V6
Nfe2.Yale.K562.Nucleolus.Total
W6
Nfe2.Yale.K562.Nucleoplasm.Total
X6
NfkbIggrab.Yale.Gm12878.Cytosol.Longnonpolya
Y6
NfkbIggrab.Yale.Gm12878.Nucleolus.Total
Z6
NfkbTnfa.Yale.Gm12878.Cytosol.Longnonpolya
A7
NfkbTnfa.Yale.Gm12878.Nucleolus.Total
B7
Nfya.Yale.K562.Cytosol.Longnonpolya
C7
Nfya.Yale.K562.Nucleolus.Total
D7
Nfya.Yale.K562.Nucleoplasm.Total
E7
Nfyb.Yale.K562.Cytosol.Longnonpolya
F7
Nfyb.Yale.K562.Nucleolus.Total
G7
Nfyb.Yale.K562.Nucleoplasm.Total
H7
Nrsf.Hudsonalpha.Gm12878.Cytosol.Longnonpolya
I7
Nrsf.Hudsonalpha.Gm12878.Nucleolus.Total
Average signal
Position relative TSS (bp)
−1000
−500
0
500
1000
Bidirectional genes
Unidirectional genes

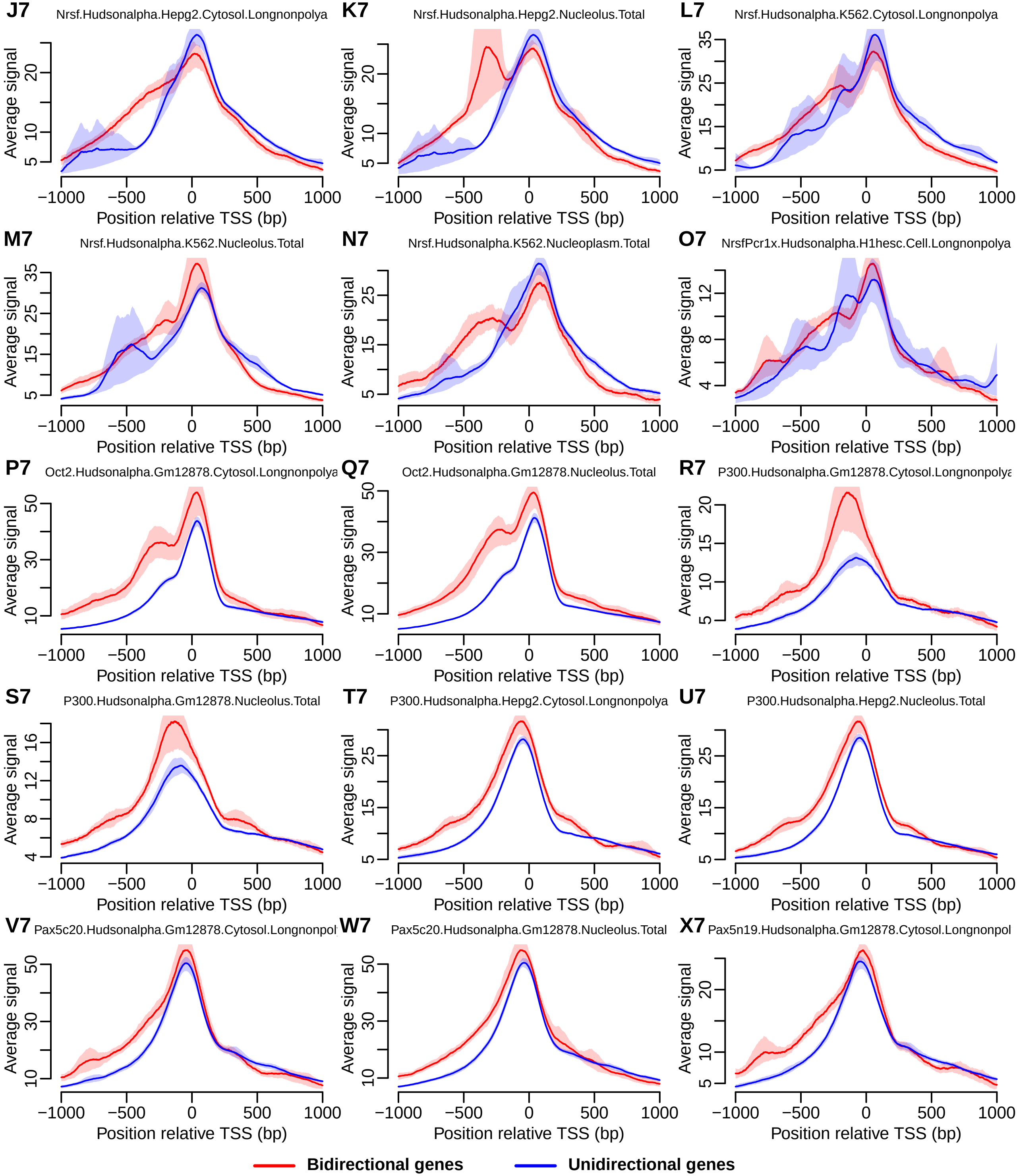
J7
Nrsf.Hudsonalpha.Hepg2.Cytosol.Longnonpolya
K7
Nrsf.Hudsonalpha.Hepg2.Nucleolus.Total
L7
Nrsf.Hudsonalpha.K562.Cytosol.Longnonpolya
M7
Nrsf.Hudsonalpha.K562.Nucleolus.Total
N7
Nrsf.Hudsonalpha.K562.Nucleoplasm.Total
O7
NrsfPcr1x.Hudsonalpha.H1hesc.Cell.Longnonpolya
P7
Oct2.Hudsonalpha.Gm12878.Cytosol.Longnonpolya
Q7
Oct2.Hudsonalpha.Gm12878.Nucleolus.Total
R7
P300.Hudsonalpha.Gm12878.Cytosol.Longnonpolya
S7
P300.Hudsonalpha.Gm12878.Nucleolus.Total
T7
P300.Hudsonalpha.Hepg2.Cytosol.Longnonpolya
U7
P300.Hudsonalpha.Hepg2.Nucleolus.Total
V7
Pax5c20.Hudsonalpha.Gm12878.Cytosol.Longnonpol
W7
Pax5c20.Hudsonalpha.Gm12878.Nucleolus.Total
X7
Pax5n19.Hudsonalpha.Gm12878.Cytosol.Longnonpol
Average signal
Position relative TSS (bp)
−1000
−500
0
500
1000
Bidirectional genes
Unidirectional genes

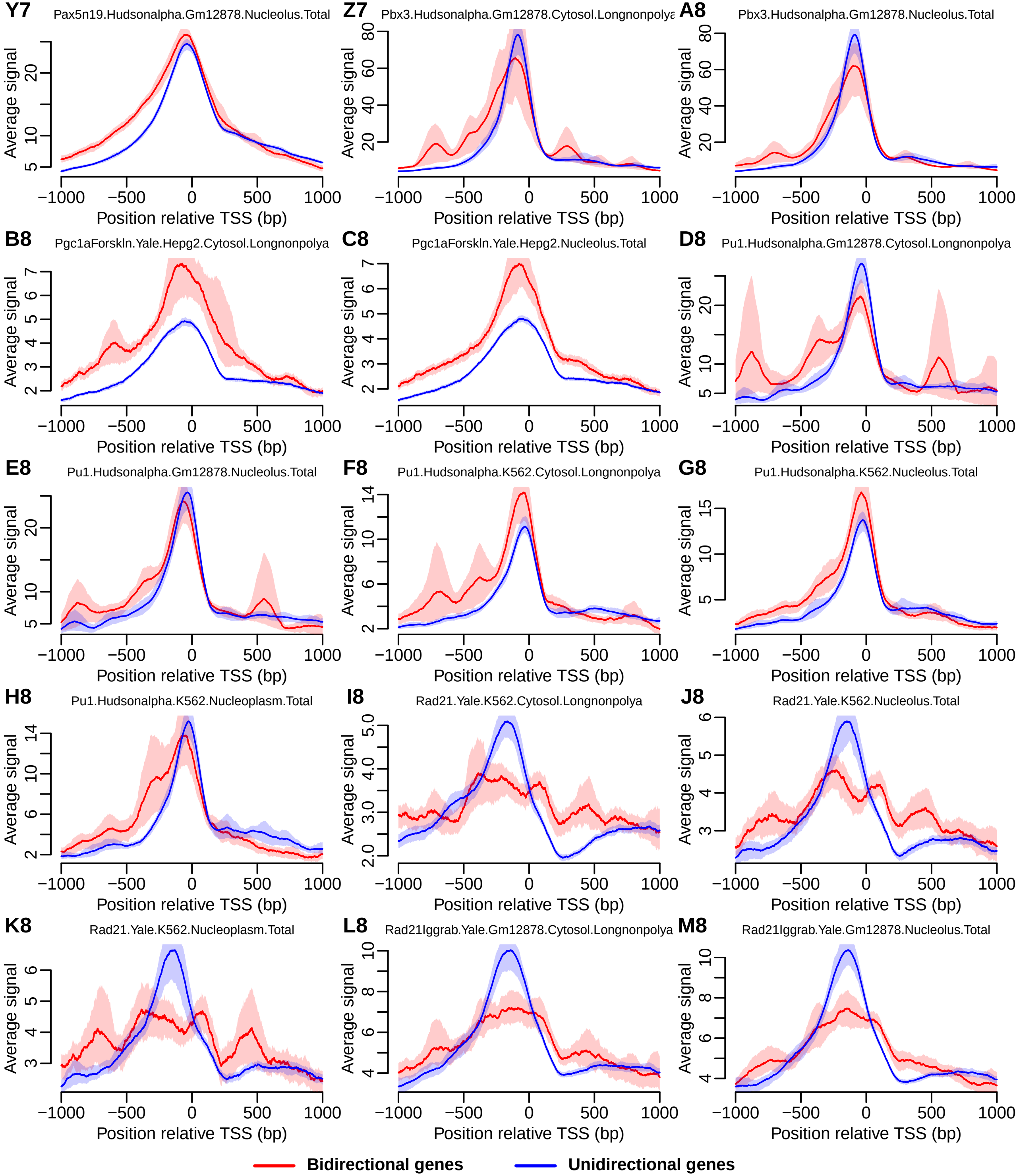

Y7
Pax5n19.Hudsonalpha.Gm12878.Nucleolus.Total
Z7
Pbx3.Hudsonalpha.Gm12878.Cytosol.Longnonpolya
A8
Pbx3.Hudsonalpha.Gm12878.Nucleolus.Total
B8
Pgc1aForskln.Yale.Hepg2.Cytosol.Longnonpolya
C8
Pgc1aForskln.Yale.Hepg2.Nucleolus.Total
D8
Pu1.Hudsonalpha.Gm12878.Cytosol.Longnonpolya
E8
Pu1.Hudsonalpha.Gm12878.Nucleolus.Total
F8
Pu1.Hudsonalpha.K562.Cytosol.Longnonpolya
G8
Pu1.Hudsonalpha.K562.Nucleolus.Total
H8
Pu1.Hudsonalpha.K562.Nucleoplasm.Total
I8
Rad21.Yale.K562.Cytosol.Longnonpolya
J8
Rad21.Yale.K562.Nucleolus.Total
K8
Rad21.Yale.K562.Nucleoplasm.Total
L8
Rad21Iggrab.Yale.Gm12878.Cytosol.Longnonpolya
M8
Rad21Iggrab.Yale.Gm12878.Nucleolus.Total
Average signal
Position relative TSS (bp)
−1000
−500
0
500
1000
Bidirectional genes
Unidirectional genes

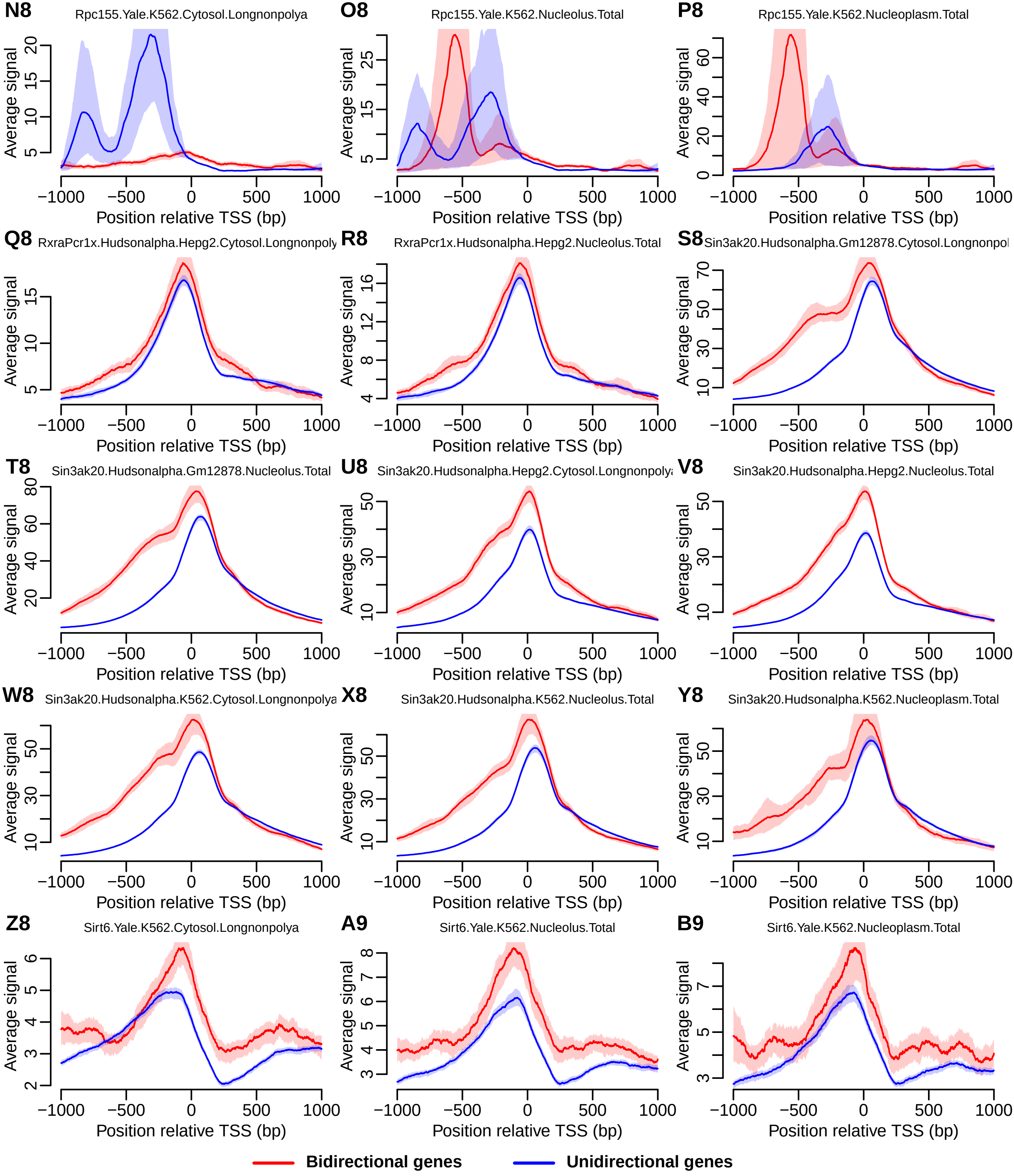
N8
Rpc155.Yale.K562.Cytosol.Longnonpolya
O8
Rpc155.Yale.K562.Nucleolus.Total
P8
Rpc155.Yale.K562.Nucleoplasm.Total
Q8
RxraPcr1x.Hudsonalpha.Hepg2.Cytosol.Longnonpoly
R8
RxraPcr1x.Hudsonalpha.Hepg2.Nucleolus.Total
S8
Sin3ak20.Hudsonalpha.Gm12878.Cytosol.Longnonpol
T8
Sin3ak20.Hudsonalpha.Gm12878.Nucleolus.Total
U8
Sin3ak20.Hudsonalpha.Hepg2.Cytosol.Longnonpoly
V8
Sin3ak20.Hudsonalpha.Hepg2.Nucleolus.Total
W8
Sin3ak20.Hudsonalpha.K562.Cytosol.Longnonpolya
X8
Sin3ak20.Hudsonalpha.K562.Nucleolus.Total
Y8
Sin3ak20.Hudsonalpha.K562.Nucleoplasm.Total
Z8
Sirt6.Yale.K562.Cytosol.Longnonpolya
A9
Sirt6.Yale.K562.Nucleolus.Total
B9
Sirt6.Yale.K562.Nucleoplasm.Total
Average signal
Position relative TSS (bp)
−1000 −500 0 500 1000
Bidirectional genes
Unidirectional genes

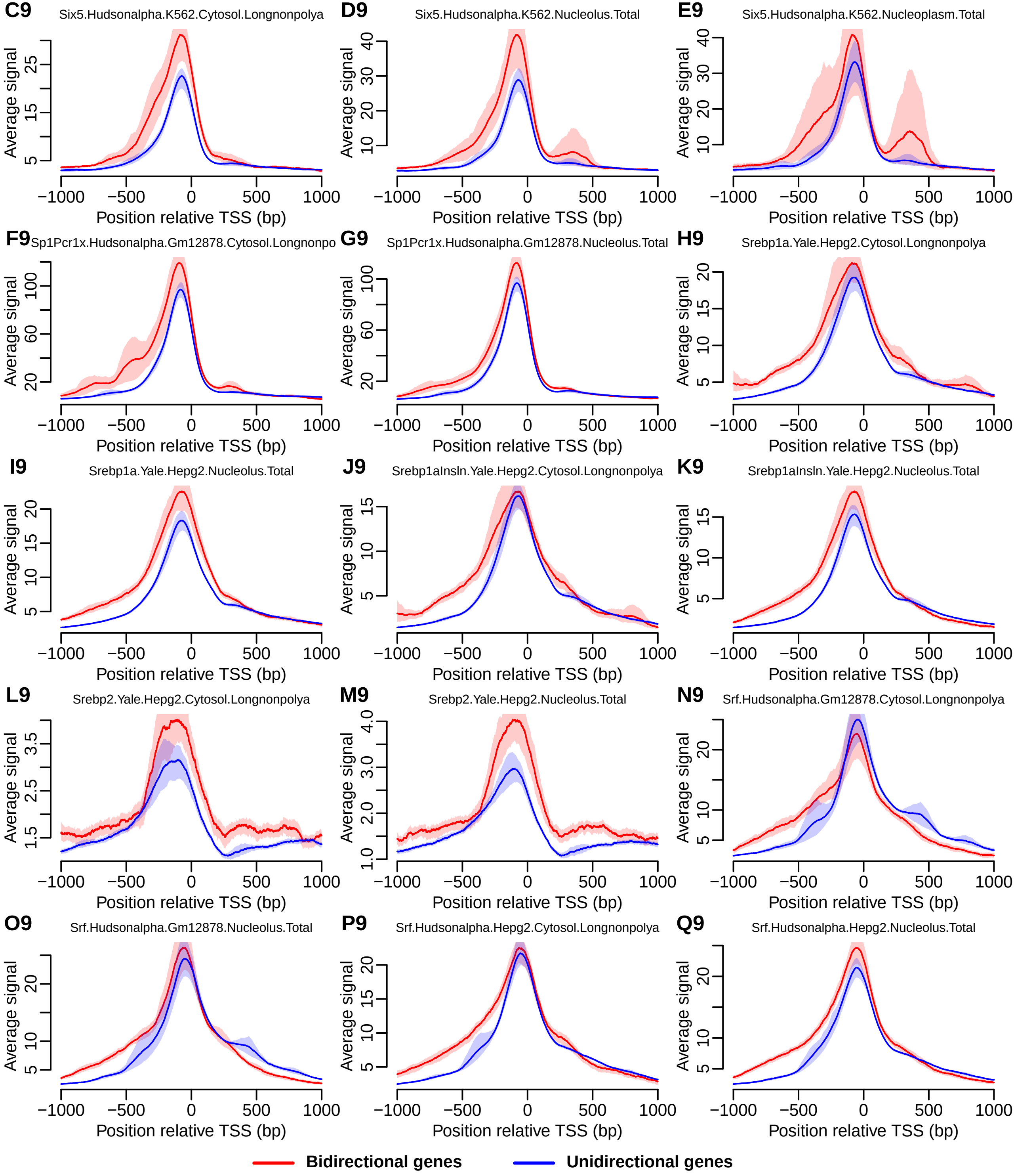
C9
Six5.Hudsonalpha.K562.Cytosol.Longnonpolya
D9
Six5.Hudsonalpha.K562.Nucleolus.Total
E9
Six5.Hudsonalpha.K562.Nucleoplasm.Total
F9
Sp1Pcr1x.Hudsonalpha.Gm12878.Cytosol.Longnonpo
G9
Sp1Pcr1x.Hudsonalpha.Gm12878.Nucleolus.Total
H9
Srebp1a.Yale.Hepg2.Cytosol.Longnonpolya
I9
Srebp1a.Yale.Hepg2.Nucleolus.Total
J9
Srebp1aInsln.Yale.Hepg2.Cytosol.Longnonpolya
K9
Srebp1aInsln.Yale.Hepg2.Nucleolus.Total
L9
Srebp2.Yale.Hepg2.Cytosol.Longnonpolya
M9
Srebp2.Yale.Hepg2.Nucleolus.Total
N9
Srf.Hudsonalpha.Gm12878.Cytosol.Longnonpolya
O9
Srf.Hudsonalpha.Gm12878.Nucleolus.Total
P9
Srf.Hudsonalpha.Hepg2.Cytosol.Longnonpolya
Q9
Srf.Hudsonalpha.Hepg2.Nucleolus.Total
Average signal
Position relative TSS (bp)
−1000
−500
0
500
1000
Bidirectional genes
Unidirectional genes

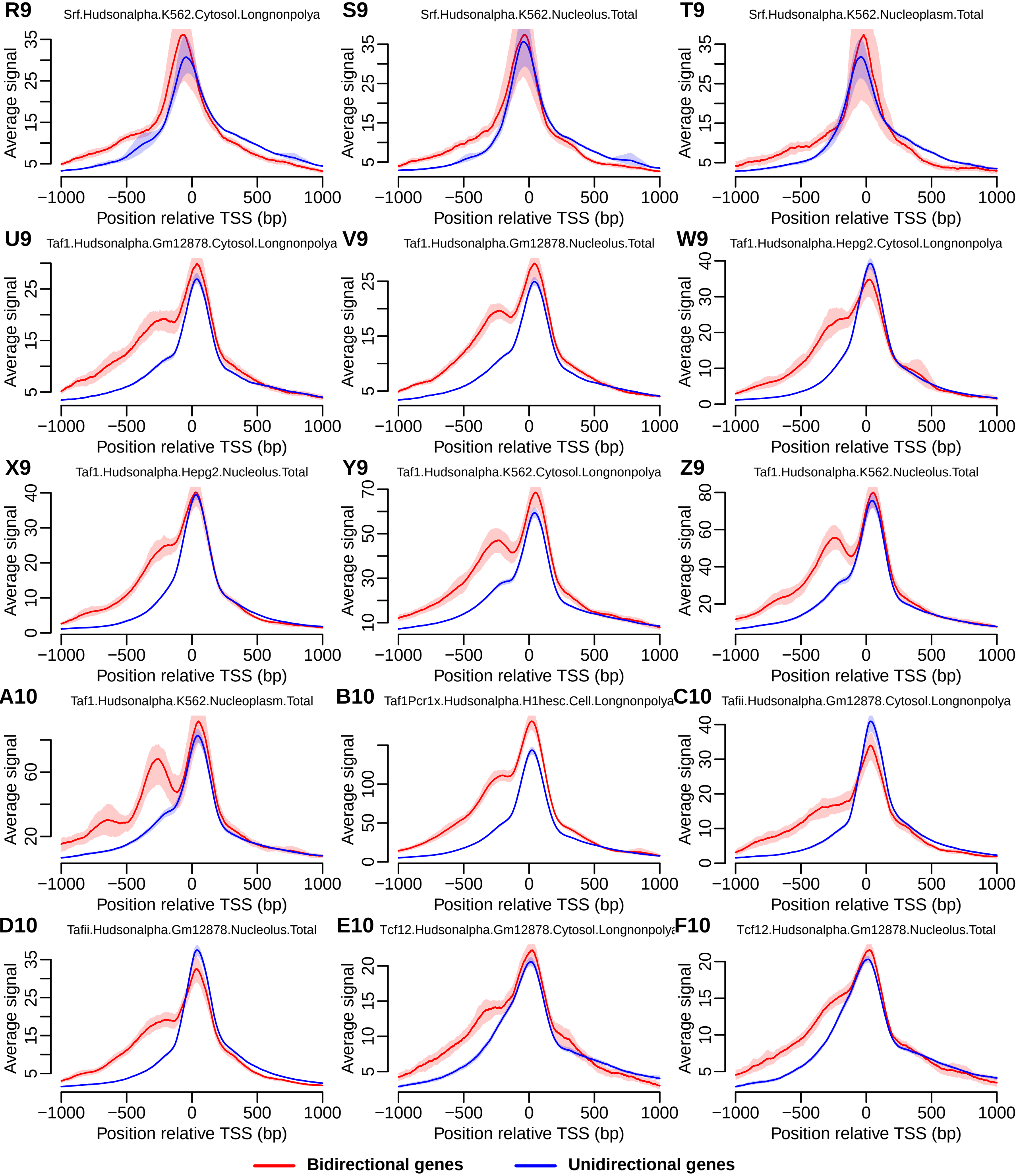
R9
Srf.Hudsonalpha.K562.Cytosol.Longnonpolya
S9
Srf.Hudsonalpha.K562.Nucleolus.Total
T9
Srf.Hudsonalpha.K562.Nucleoplasm.Total
U9
Taf1.Hudsonalpha.Gm12878.Cytosol.Longnonpolya
V9
Taf1.Hudsonalpha.Gm12878.Nucleolus.Total
W9
Taf1.Hudsonalpha.Hepg2.Cytosol.Longnonpolya
X9
Taf1.Hudsonalpha.Hepg2.Nucleolus.Total
Y9
Taf1.Hudsonalpha.K562.Cytosol.Longnonpolya
Z9
Taf1.Hudsonalpha.K562.Nucleolus.Total
A10
Taf1.Hudsonalpha.K562.Nucleoplasm.Total
B10
Taf1Pcr1x.Hudsonalpha.H1hesc.Cell.Longnonpolya
C10
Tafii.Hudsonalpha.Gm12878.Cytosol.Longnonpolya
D10
Tafii.Hudsonalpha.Gm12878.Nucleolus.Total
E10
Tcf12.Hudsonalpha.Gm12878.Cytosol.Longnonpolya
F10
Tcf12.Hudsonalpha.Gm12878.Nucleolus.Total
Average signal
Position relative TSS (bp)
−1000
−500
0
500
1000
Bidirectional genes
Unidirectional genes

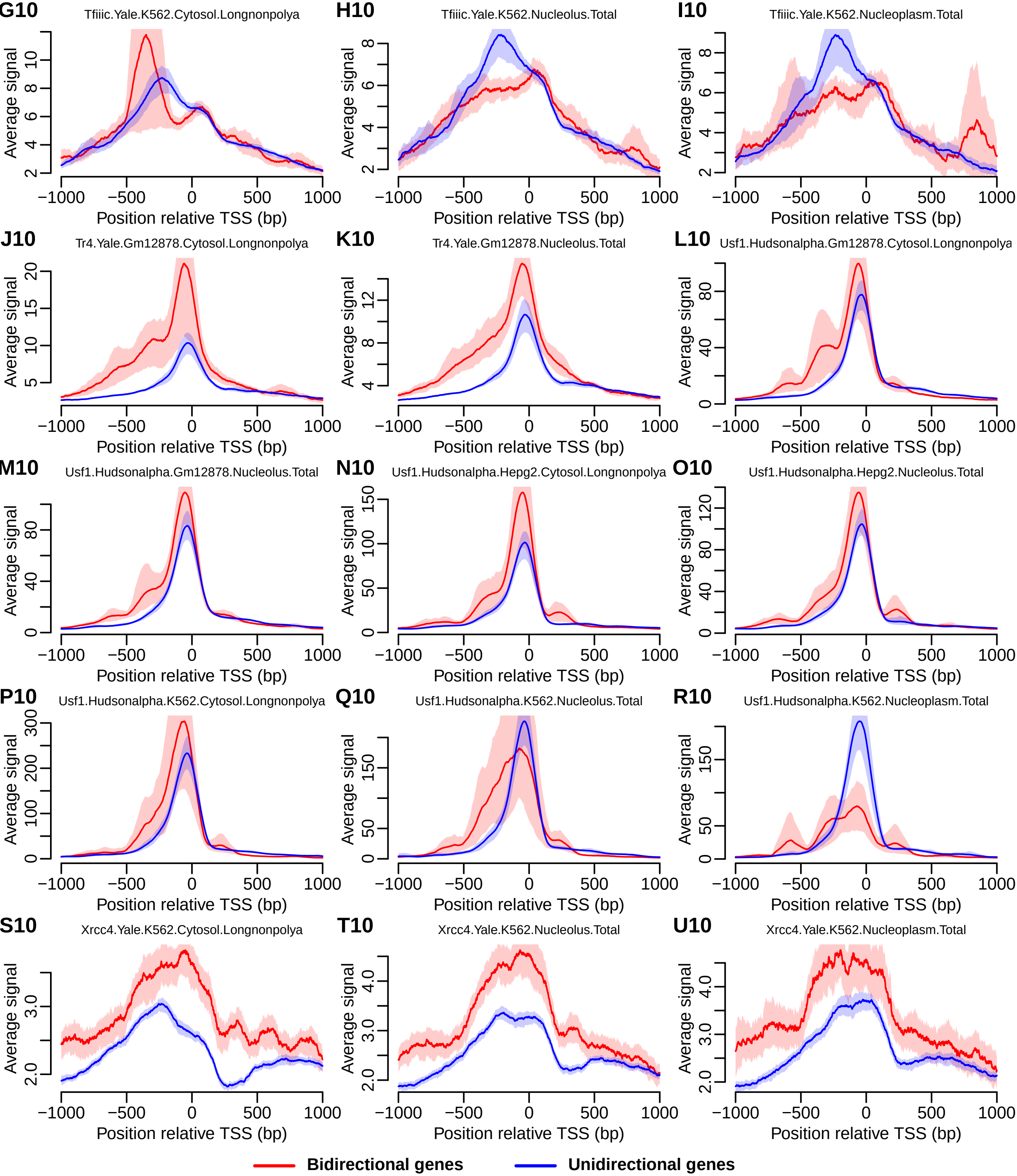

G10
Tfiiic.Yale.K562.Cytosol.Longnonpolya
H10
Tfiiic.Yale.K562.Nucleolus.Total
I10
Tfiiic.Yale.K562.Nucleoplasm.Total
J10
Tr4.Yale.Gm12878.Cytosol.Longnonpolya
K10
Tr4.Yale.Gm12878.Nucleolus.Total
L10
Usf1.Hudsonalpha.Gm12878.Cytosol.Longnonpolya
M10
Usf1.Hudsonalpha.Gm12878.Nucleolus.Total
N10
Usf1.Hudsonalpha.Hepg2.Cytosol.Longnonpolya
O10
Usf1.Hudsonalpha.Hepg2.Nucleolus.Total
P10
Usf1.Hudsonalpha.K562.Cytosol.Longnonpolya
Q10
Usf1.Hudsonalpha.K562.Nucleolus.Total
R10
Usf1.Hudsonalpha.K562.Nucleoplasm.Total
S10
Xrcc4.Yale.K562.Cytosol.Longnonpolya
T10
Xrcc4.Yale.K562.Nucleolus.Total
U10
Xrcc4.Yale.K562.Nucleoplasm.Total
Average signal
Position relative TSS (bp)
−1000 −500 0 500 1000
Bidirectional genes
Unidirectional genes

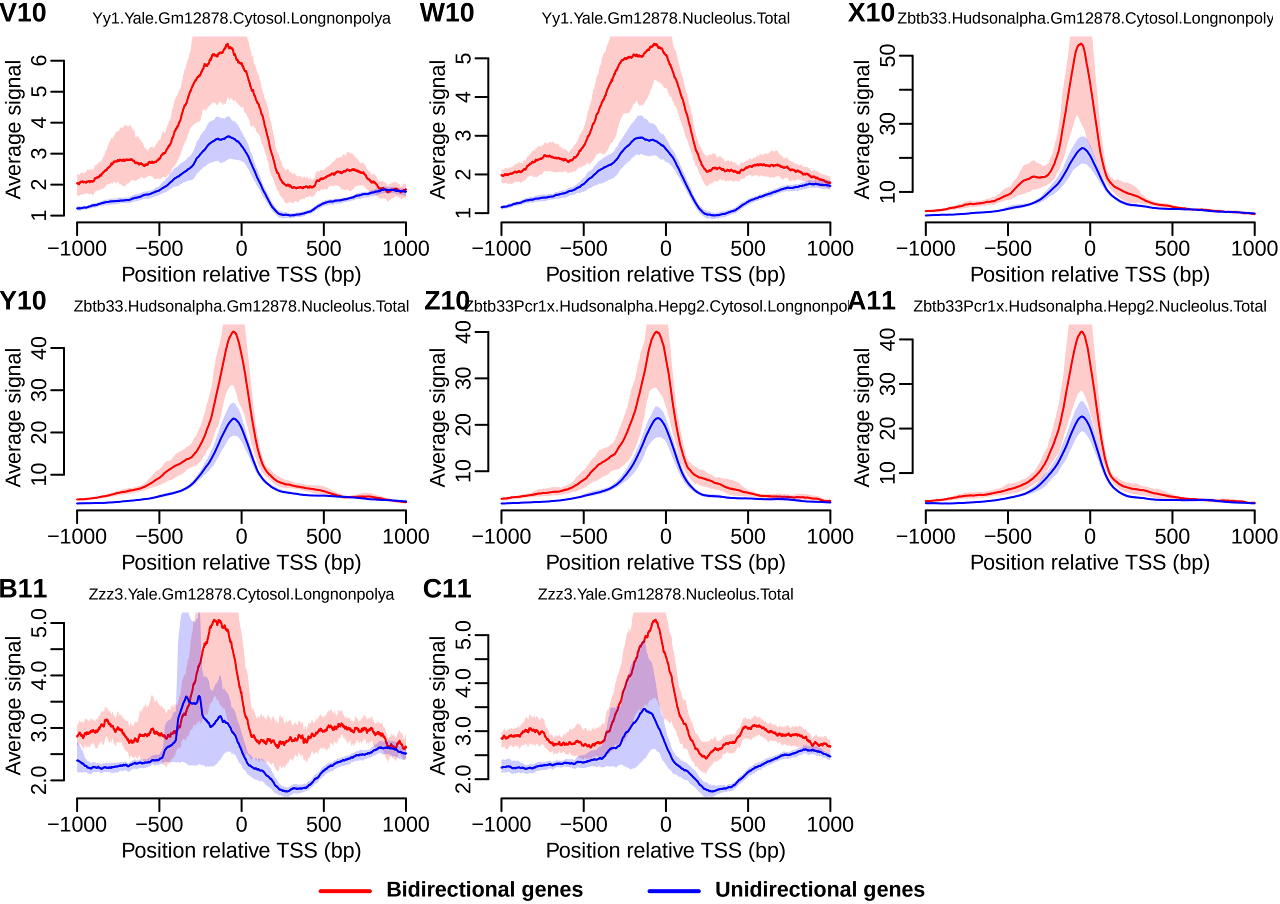
V10
Yy1.Yale.Gm12878.Cytosol.Longnonpolya
W10
Yy1.Yale.Gm12878.Nucleolus.Total
X10
Zbtb33.Hudsonalpha.Gm12878.Cytosol.Longnonpoly
Y10
Zbtb33.Hudsonalpha.Gm12878.Nucleolus.Total
Z10
Zbtb33Pcr1x.Hudsonalpha.Hepg2.Cytosol.Longnonpol
A11
Zbtb33Pcr1x.Hudsonalpha.Hepg2.Nucleolus.Total
B11
Zzz3.Yale.Gm12878.Cytosol.Longnonpolya
C11
Zzz3.Yale.Gm12878.Nucleolus.Total
Average signal
Position relative TSS (bp)
−1000
−500
0
500
1000
Bidirectional genes
Unidirectional genes
